# Novel Drug Design for Treatment of COVID-19: A Systematic Review of Preclinical Studies

**DOI:** 10.1155/2022/2044282

**Published:** 2022-09-25

**Authors:** Sarah Mousavi, Shima Zare, Mahmoud Mirzaei, Awat Feizi

**Affiliations:** ^1^Department of Clinical Pharmacy and Pharmacy Practice, School of Pharmacy and Pharmaceutical Sciences, Isfahan University of Medical Sciences, Isfahan, Iran; ^2^School of Pharmacy and Pharmaceutical Sciences, Isfahan University of Medical Sciences, Isfahan, Iran; ^3^Child Growth and Development Research Center, Research Institute for Primordial Prevention of Non-Communicable Disease, Isfahan University of Medical Sciences, Isfahan, Iran; ^4^Department of Epidemiology and Biostatistics, School of Health, Isfahan University of Medical Sciences, Isfahan, Iran

## Abstract

**Background:**

Since the beginning of the novel coronavirus (SARS-CoV-2) disease outbreak, there has been an increasing interest in discovering potential therapeutic agents for this disease. In this regard, we conducted a systematic review through an overview of drug development (in silico, in vitro, and in vivo) for treating COVID-19.

**Methods:**

A systematic search was carried out in major databases including PubMed, Web of Science, Scopus, EMBASE, and Google Scholar from December 2019 to March 2021. A combination of the following terms was used: coronavirus, COVID-19, SARS-CoV-2, drug design, drug development, In silico, In vitro, and In vivo. A narrative synthesis was performed as a qualitative method for the data synthesis of each outcome measure.

**Results:**

A total of 2168 articles were identified through searching databases. Finally, 315 studies (266 in silico, 34 in vitro, and 15 in vivo) were included. In studies with in silico approach, 98 article study repurposed drug and 91 studies evaluated herbal medicine on COVID-19. Among 260 drugs repurposed by the computational method, the best results were observed with saquinavir (*n* = 9), ritonavir (*n* = 8), and lopinavir (*n* = 6). Main protease (*n* = 154) following spike glycoprotein (*n* = 62) and other nonstructural protein of virus (*n* = 45) was among the most studied targets. Doxycycline, chlorpromazine, azithromycin, heparin, bepridil, and glycyrrhizic acid showed both in silico and in vitro inhibitory effects against SARS-CoV-2.

**Conclusion:**

The preclinical studies of novel drug design for COVID-19 focused on main protease and spike glycoprotein as targets for antiviral development. From evaluated structures, saquinavir, ritonavir, eucalyptus, Tinospora cordifolia, aloe, green tea, curcumin, pyrazole, and triazole derivatives in in silico studies and doxycycline, chlorpromazine, and heparin from in vitro and human monoclonal antibodies from in vivo studies showed promised results regarding efficacy. It seems that due to the nature of COVID-19 disease, finding some drugs with multitarget antiviral actions and anti-inflammatory potential is valuable and some herbal medicines have this potential.

## 1. Introduction

Coronavirus disease 2019 (COVID-19), which was first identified in December 2019, and shortly after, declared a pandemic by World Health Organization (WHO) [1]. As of January 18, 2022, there have been more than 326 million confirmed cases and 5.54 million deaths globally [2]. Coronaviruses belong to the family of Coronaviridae, RNA viruses with crown-like spikes on the surface of the coronavirus particles. According to a meta-analysis of Macedo et al. [3], the mortality rate of COVID-19 was 17.1% for patients admitted to hospitals, whereas WHO estimated a fatality rate of 6.73%, which was much lower than that calculated from published studies. Among the critical cases of COVID-19, the mortality rate reaches 40% [4].

Substantial efforts have been made in the treatment of patients with COVID-19. The WHO recommendations in the treatment of COVID-19 are as follows [5]: molnupiravir (conditional), baricitinib (strong), ruxolitinib and tofacitinib (conditional), sotrovimab (conditional), casirivimab and imdevimab (conditional), IL-6 receptor blockers (tocilizumab and sarilumab) (strong), remdesivir (conditional), and systemic corticosteroids (strong). The WHO recommends not to use ivermectin, lopinavir/ritonavir, hydroxychloroquine, and convalescent plasma.

The pathogenesis of COVID-19 was explained by cytokine storm, reduction in ACE2 expression, and activation of complement pathway-induced microvascular injury and thrombosis [6]. The mechanisms of the recommended agents are focused on the mentioned pathogenesis to improve the clinical outcome of COVID-19, and antiviral therapies are missing. The anticoronaviral strategies include preventing the synthesis of viral RNA, inhibiting virus replication, blocking the virus binding to human cell receptors, or inhibiting the viruses' self-assembly process [7]. The SARS-CoV-2 contains at least four structural proteins: spike (S) protein, envelope (E) protein, membrane (M) protein, and nucleocapsid (N) protein, and 16 nonstructural proteins (NSPs). Among the translated NSPs, the main protease, also called chymotrypsin-like protease (3C-like protease), and the papain-like protease are two essential proteases for proteolytic processing of the coronavirus replicase polyprotein, therefore generating functional replication complex of the virus, whereas RNA-dependent RNA polymerase is the central enzyme for RNA synthesis. These three NSPs play crucial roles in coronavirus replication, making them attractive targets for anticoronaviral drug design [8].

The S protein, a surface-located trimeric glycoprotein of coronaviruses, promotes the attachment of viruses to host cells through binding to angiotensin-converting enzyme 2 (ACE2) and virus-cell membrane fusion during viral infection. Thus, the S protein has been considered as a major target for the development of vaccines and drug [9].

The development of a new therapeutic agent is a complex, lengthy, and expensive process, which can take 2–4 years of preclinical development and 3–6 years of clinical development and over 500 million dollar cost. There are three critical steps to develop a new drug including discovery and development, preclinical research, and clinical development [10].

Drug discovery involves screening hits, medicinal chemistry, and optimization of hits to reduce potential drug side effects. For drug discovery, two different complementary approaches can be applied: classical pharmacology, also known as phenotypic drug discovery, which is the historical basis of drug discovery, and reverse pharmacology, also known as designated target-based drug discovery. Screening methods based on phenotypic drug discovery have been used to discover new natural products mainly from the terrestrial origin [11]. These two strategies have advantages and disadvantages and promote very different screening assays. The frequent re-discovery of the same compounds, the technical difficulties associated with the isolation of compounds from extracts, and the incompatibility of natural product extracts with high-throughput screening (HTS) campaigns were the disadvantages of phenotypic drug discovery. On the other hand, natural product structures have the characteristics of high chemical diversity, biochemical specificity, and other molecular properties that make them favorable as lead structures for drug discovery, which serve to differentiate them from libraries of synthetic and combinatorial compounds [12]. Overly simplified assays, acting of drug on more than one target, the multifactorial nature of diseases, and challenges to identify a single molecular target are some limitations of target-based drug discovery. Therefore, a comprehensive screening strategy will incorporate both targeted and phenotypic assays, with one format designated as the primary screen and the other as a secondary or follow-up assay. During the spread of COVID-19 outbreak, great efforts have been made in therapeutic drug discovery against the virus. Because COVID-19 is a new, acute, severe infectious disease, the anti-SARS-CoV-2 drug development strategies are to screen existing drugs to identify potentially effective drugs, to expand indications, and to develop a vaccine [12]. The safety of conventional drugs has been mostly verified; if effective, they can be quickly applied in clinical practice (repurposing of existing drugs). The recent rises of several high transmissible strains sounded alarms for currently used vaccines and drugs. Therefore, developing broad-spectrum antiviral drugs not only to combat COVID-19 but also to provide protective arsenals against future viral outbreaks is a requirement. Scientists continue the development of broad-spectrum antiviral drugs from natural or chemical sources, which have the potential advantages of broad-spectrum therapeutic effect and insensitivity to viral evasion. Given the urgency of the SARS-CoV-2 outbreak, here we discuss the discovery and development of new therapeutics for SARS-CoV-2 infection based on the strategies from preclinical drug discovery.

## 2. Methods

We report this systematic review based on the Preferred Reporting Items for Systematic Reviews and Meta-Analysis (PRISMA) guidelines [13].

### 2.1. Data Sources and Searches

Studies published in PubMed, Scopus, Web of Science, EMBASE, Google Scholar, and DrugBank were searched from December 2019 to March 2021 using the following search terms: “Coronavirus,” “Covid-19,” “SARS-CoV-2,” “Drug design,” “Drug development,” “In silico,” “In vitro,” and “In vivo” alone or in combination without language restrictions. The keywords were selected using expert opinions, mesh, and related article titles.

All articles with full text or in the absence of full text with abstract are included in the screening of this study. Studies were excluded if studies were comments, editorial, letters, review, and preprints.

### 2.2. Data Extraction

Two researchers independently extracted data from included studies using a predefined data extraction form. All disagreement was discussed and solved after rechecking the source data with a third investigator. The data extracted, including the last name of the first author, type of the study (in silico, in vitro, in vivo), and name of the agent (chemical compound, drug, herb, etc.), studied the mechanism and efficacy of the agent according to study design that specified according to the following definition.

Computer-aided drug design can be divided into three different categories. All are based on ligands and receptors, which are briefly described [14] as follows:

1. *Dock Receptor-Based Approach* [15]. Once the three-dimensional structure of the ligand molecules and their receptor is known, the receptor-based method is a good candidate for identifying or optimizing drugs. Due to the presence of three-dimensional structures of compounds and receptors, the nature of the interaction between the ligand and the receptor and the type of structure that the ligand can have to interact with them in favorable conditions can be identified using this method. The compound is simulated on the active site of the dock (meaning anchoring) and on the interaction of the ligand with the receptor by molecular mechanics and molecular dynamics. In this method, due to the ligation of the ligand in the active position, the ligand changes in terms of conformity and changes its position in different conditions and shows interaction with the receptor in different types of situations. To determine the type of ligands that can be docked into the receptor site, the matching of the shape and the complementarity of the hydrophobic, hydrophilic, and charged parts must be considered. Various software packages such as AUTODOCK-Glide-LUDI and LigandFit are used to design the drug based on the structure of the receptor.
2. *Ligand-Based Approach* [16]. This method is used where the three-dimensional structures of the receptor are unknown and instead the structure of the ligands is known, which is one of the common methods. In this method, by indirectly studying compounds that react with biomolecules, they seek to design compounds that are pharmacologically active. In ligand-based drug design methods, in the absence of biomolecule structure, by studying specific ligands, it seeks to identify the structural and physicochemical properties of the compounds so that the desired compound can be designed based on data extracted from the study of previous compounds. This method is a kind of drug design based on pharmacophore (pharmacophore refers to the part of the drug to which the effect of the drug depends on that part of the molecule), and by studying the quantitative relationship between structure and their activity, drugs can be designed by this method. It can be said that it is a method for designing the pharmacophores of drugs.
3. *Denovo Design-Based Approach* [17]. This method is used when the structure of the ligand is unknown but the structure of the receptor is known. In this method, there is information about the structures of the receptor or quasi-receptors, but there is no structure of the main composition that can interact with the active site of the receptor. One of the functions of drug design based on this method is to suggest and present the main composition that is complementary to the active site. The basis of the method is that the database of existing 3D structures is used to find small molecules that can interact with the active site of the receptor in terms of size, geometry, and functional groups. Software packages such as GROW and LEGEND are used to design drugs by this method.

**Figure 1:**
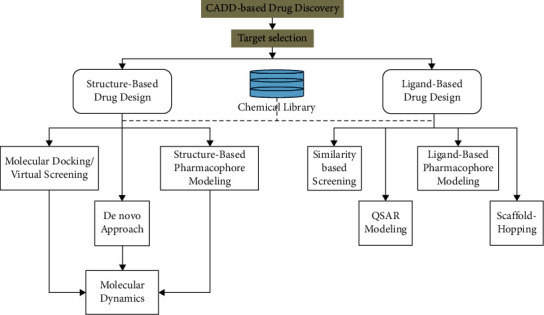
Computational methods in drug design (CADD: computer-aided drug design).

Drug design methods in the computer include quantitative structure-activity relationship (RASQ), docking, molecular dynamic simulation, and computational modeling. In these studies, the efficacy is evaluated based on the function of the drug or compound agent and the mechanism of action.  Figure 1 shows the methods of computer-aided drug design (CADD).

#### 2.2.1. Computational Methods in Drug Design

Quantitative Structure-Activity Relationship (QSAR). QSAR provides studies on the relationship between chemical structure and biological activity or other biological activities that are important in selecting or removing a compound before synthesis and testing. QSAR [18] is especially important to predict the result, especially when it is not possible to experiment with a compound. Molecular descriptors, which are the most important components of QSAR, can be obtained experimentally or through mathematical formulas from various theories such as quantum mechanics, chemical graph theory, and study theories. QSAR seeks to establish a statistically significant relationship between structure and performance. It also explains the specific effect of a drug and can ultimately predict the effect of newly synthesized chemical compounds. QSAR model is also an equation that predicts a property through molecular descriptors and their coefficients. Evaluation of the effectiveness of new compounds that have been studied using this method can be reported as a percentage of enzyme inhibition if the modeling has been done and mentioned in the article.

#### 2.2.2. Docking

In this technique, to achieve a combination with a pharmacological effect and increase the pharmacological activity of the drug, different formulations of a drug interact with the receptor, and the structure that has the best interaction with the receptor and the lowest energy level is selected for laboratory steps [19]. In this way, possible structures that have a stronger interaction with the receiver can be isolated at this stage. The same issue is considered and reported as an effectiveness measure.

#### 2.2.3. Molecular Dynamic Simulation

In this technique, which is based on the simulation of drug-receptor interaction in the body, docking problems are solved and in fact play a complementary role in this technique [20]. Due to the time-consuming work with this technique, effective structures cannot be achieved directly through it, and the final stages of the study of drug-receptor interactions should be evaluated before starting laboratory work by having effective compounds from the previous stages. Molecular dynamic simulations produce information at the microscopic level (position and velocity of atoms). The conversion of these data into macroscopic values (pressure, energy, etc.) is done using statistical mechanics. In fact, molecular dynamics and statistical mechanics link microscopic concepts and macroscopically observable quantities. Molecular dynamic simulations are only able to predict the thermodynamic behavior and stability of the ligand binding mechanism at the active site of the target enzyme. This is reported as a criterion.

#### 2.2.4. In Vitro

Study of drug in cell culture medium: effectiveness in these studies means inhibition of the replication of COVID-19 by the compound or drug under study [21].

The half-maximal inhibitory concentrations (IC_50_) are a measure of the effectiveness of a compound in inhibiting biological function [22].

In vivo studies are those in which the effects of drugs are tested on whole living organisms or cells usually animals as opposed to a tissue organism or dead organism. In vivo testing is better studied for observing the overall effects of an experiment on living subjects [23].

## 3. Results

**Figure 2:**
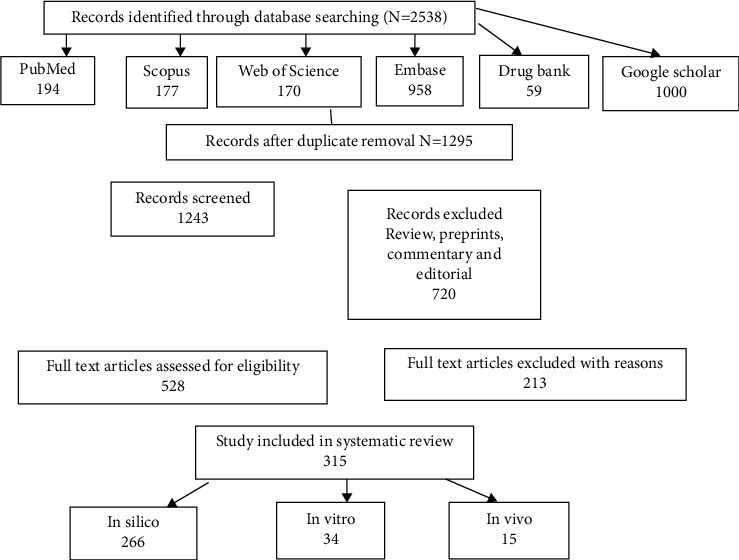
PRISMA diagram of the study.

In this review, we reported a significant number of articles with in silico, in vitro, and in vivo approaches for drug development of COVID-19. We retrieved a total of 2538 articles from the initial database search. After the removal of duplication and screening, 317 studies were selected for inclusion in this review.  Figure 2 shows the PRISMA diagram.

The analysis of article contents indicated that 266 studies performed in silico approaches against viral targets; 34 studies used in vitro approaches against SARS-CoV-2; and 15 studies used in vivo (animal) models.

### 3.1. Results from In Silico Drug Discovery

From 267 studies used in silico approaches, 98 article studies repurposed approved drugs with a new mechanism of action and 91 studies evaluated natural products (e.g., herbal medicine) on COVID-19. The characteristics of these studies are summarized in Tables1 and 2. Also, Table 3 shows the characteristics of the remaining studies (*N* = 87).

In silico studies used the following component of novel coronavirus as targets: main protease (*N* = 154), spike glycoprotein (*N* = 62), nonstructural protein (*N* = 45), RNA-dependent RNA polymerase (*N* = 21), papain-like protease (*N* = 19).

About 260 drugs were repurposed by the computational methods for COVID-19 therapy such as about 120 drugs candidate against the main protease, 52 drugs against the spike glycoprotein, 14 drugs against RNA-dependent RNA polymerase, and 28 drugs against other nonstructural proteins.

Among the studied repurposed drugs, the best results (regarding efficacy) were observed with saquinavir (*N* = 9 study), ritonavir (*N* = 8 study), lopinavir (*N* = 6 study), remdesivir (*N* = 3 study), and amikacin, danoprevir, favipiravir, and telaprevir.

Table 1 shows target-based synthesis of data for COVID-19 drug repurposing. As presented, at least two studies show the efficacy of aprepitant, cobicistat, dipyridamole, and dihydroergotamine against the main protease and tegobuvir (*N* = 2) against spike protein. The following list of drugs had multitarget action: avapritinib, famotidine, bictegravir, ziprasidone, capmatinib, pexidartinib, amprenavir, zafirlukast, cilostazol, paromomycin, lopinavir, and remdesivir.

A total of 91 studies used in silico methods to evaluate the effects of natural products including herbal medicine against SARS-CoV-2. Among them, 54 studies used the main protease as main target, in which eucalyptus (*N* = 3), Tinospora cordifolia (*N* = 3), and flavonoids (e.g., hesperidin, rutin, and herbacetin) were the most studied and effective. Other studied targets were as follows: spike (*N* = 22) and multitarget (*N* = 20). From the plant metabolites, oleanolic acid, hesperidin, epigallocatechin gallate, jensenone, tinosponone, and anistone show promising results in computational methods against COVID-19. Aloe, green tea, eucalyptus, curcumin, and many Chinese, Indian, and African plants were also effective on COVID-19 in silico.

The derivatives of pyrazoles, oxadiazoles, phenyltriazolinones, triazoles, benzoylpinostrobin, benzoic acid, benzylidenechromanones, coumarin, and selenium show efficacy in computational methods and could be considered as lead molecules for drug design and synthesis against COVID-19. Melatonin, ebselen, phenyl furoxan, thimerosal, isatin, romidepsin, phenyl mercurin, pleconaril, and tyrosine kinase inhibitors (such as nilotinib and imatinib) are examples of other drugs that are effective in in silico methods.

### 3.2. Results from In Vitro and In Vivo Studies

Screening of in vitro studies leads to finding 34 studies. Different compounds were evaluated and most studies focused on the inhibition of viral replication, which was assessed by the quantification of viral RNA by PCR and IC_50_ value reported. Most of the studies use the Vero E6 cell line for the assessment of replication of SARS-CoV-2 (Table 4). In particular, main protease, papain-like protease, RNA-dependent RNA polymerase, NSP-14, NSP-15, spike protein, and TMPRSS2 were evaluated in vitro as targets. Also, three studies focused on in vitro inhibition of inflammatory markers such as interleukins and the effects of the cytopathic effect on infected cell.

Nitric oxide, ginkgolic acid, anacardic acid, troxerutin, bisindolylmaleimide derivatives, small molecules (GRL-172, and 5 h), baicalin and baicalein (phytochemicals), and bepridil were effective drugs in vitro against the main protease of COVID-19.

The following drugs could inhibit the spike/ACE2-mediated cell entry of COVID-19 in vitro: romidepsin, panobinostat, givinostat, sirtinol, saquinavir, lipopeptides, hydroxyzine, azelastine, heparin, and glycyrrhizic acid.

Alkenyl sulfonylurea derivatives [7], an IMU-8381 inhibitor of human dihydroorotate dehydrogenase, pyrazole derivatives, and phillyrin, regulated the expression of inflammatory cytokines (e.g., IL, TNF-*α*, and NF-*κ*B) induced by SARS-CoV-2 markedly and could be considered as adjuvant treatment of COVID-19 severe disease.

Doxycycline, chlorpromazine, azithromycin, heparin, bepridil, tannic acid, and glycyrrhizic acid are well-known drugs that show both in silico and in vitro inhibitory effects against SARS-CoV-2 and should be considered for this purpose.

Also, in vitro studies show that lopinavir/ritonavir, sofosbuvir, and favipiravir have no antiviral effects against SARS-CoV-2 (huge gap between in vitro IC_50_ and free plasma concentration).

We included 16 in vivo studies in our final analysis. Most of them use a combination of in vitro and in vivo methods for the evaluation of novel drugs.  Table 5 shows the detail of these studies.

Human monoclonal antibodies were the most evaluated drugs (*N* = 4), promote the reduction in viral load (in vitro), and prevent infection in animal models of SARS-CoV-2.

A prodrug of hydroxycytidine (molnupiravir) improved pulmonary function and reduced viral titer in vivo and was introduced as a potential broad-spectrum antiviral agent against SARS-CoV-2.

Chloroquine and chlorpromazine did not inhibit viral replication in mouse lungs, but protected them against clinical disease.

Dalbavancin shows significant inhibitory ability in both in vitro and in vivo models of COVID-19. The drug binds directly to ACE2 and blocks its interaction with the SARS-CoV-2 spike protein.

## 4. Discussion

Drug development is a multistep process, typically requiring more than five years to assure the safety and efficacy of the new compound. There are several strategies in antiviral drugs for coronaviruses including empirical testing of known antiviral drugs, large-scale phenotypic screening of compound libraries, and target-based drug discovery. To date, an increasing number of drugs have been shown to have anticoronavirus activities in vitro and in vivo, but only remdesivir and several neutralizing antibodies have been approved by the US FDA for treating COVID-19. However, remdesivir's clinical effects are controversial and new antiviral drugs are still urgently needed. Given the urgency of the SARS-CoV-2 outbreak, here we discuss the discovery and development of new therapeutics for SARS-CoV-2 infection, which have been conducted in basic research (in silico) and preclinical study (in vitro, in vivo). Our database search identified about 3000 studies, which means a global effort for drug development in the current COVID-19 pandemic. Although the chance of successful drug development is very low (less than about 10%) and till today, there are no approved drugs. Many potential candidates (at least 420 drugs) are in clinical trials.

As summarized in the results, many compounds are in the development process for COVID-19 disease. Some of these compounds are completely new and could serve as seeds (or leads) for developing antiviral drugs against COVID-19, but as we need therapeutics as soon as possible, half of the studies focused on drug repurposing (repositioning), which is a process of investigation of existing drugs for new therapeutic purposes. With the emergence of a growing COVID-19 pandemic, the drug repurposing process was being accelerated. Clinical trials using repurposed drugs may take less time and have a lower overall cost of manufacturing and could have a wide distribution of drugs. According to our results, 260 drugs repurposed by the computational methods for COVID-19, among them saquinavir, ritonavir, and lopinavir, showed the best efficacy in in silico environment. These drugs can be rapidly repurposed for clinic application for treating COVID-19 patients given their proven safety.

Many trials are performed using a combination of ritonavir-lopinavir. The results of a systematic review and meta-analysis showed that this drug combination has no more treatment effects than other therapeutic agents in COVID-19 patients and is currently not used anymore [134]. We could not find any clinical trial on saquinavir, which is the most studied drug in silico and show high potency against COVID-19. Saquinavir could be a suggestion for further clinical research.

Given that the development of synthetic chemicals for therapeutic use is a random process that might result in serendipitous discovery, many pharmaceutical companies are now focused on the development of plant-derived drugs. Natural products and their structural analogs have historically made a major contribution to pharmacotherapy, especially for cancer and infectious diseases. Nevertheless, natural products also present challenges for drug discovery, such as technical barriers to screening, isolation, characterization, and optimization. In recent years, several technological and scientific developments—including improved analytical tools, genome mining and engineering strategies, and microbial culturing advances—are addressing such challenges and opening up new opportunities. Consequently, interest in natural products as drug leads is being revitalized. Medicinal plants have attracted significant attention to treat infectious diseases. Complex molecular structures and a wide variety of natural compounds make medicinal plants an excellent biological resource for drug discovery. Our results show that various plants have potential antiviral activities and could use or be a basis for drug development against COVID-19.

Some of the studied plants are among what used by people on a daily basis, such as green tea, aloe, curcumin, and eucalyptus. A systematic review and meta-analysis of RCTs on herbal medicine in the management of COVID-19 show the significant effects of the combined therapy of herbal medicine in treating COVID-19 without any significant side effects [135].

Our results retrieved 91 in silico studies of natural products including herbal medicine. The results of this in silico approach showed that some of these studied active ingredients have a high affinity for each of the four important viral proteins compared with the inhibitors previously reported for each of these proteins. They could possibly have an inhibitory effect on the SARS-CoV-2 and COVID-19. Most evaluated chemical compounds had inhibitory effects against one or two proteins of SARS-CoV-2. In addition to having a great affinity to attach to the viral proteins, these herbal compounds have antioxidant, vasoprotective, anticarcinogenic, and antiviral properties. Thus, they can be applied as extremely safe therapeutic natural compounds and clinical assessments might have notable outcomes for controlling COVID-19.

Due to the nature of preclinical (in vitro and in vivo) studies, the number of drug development studies in this area was less than in silico studies. Again, repurposed drugs were the most studied drugs in vitro (both herbal and synthetic). Some of them such as saquinavir, heparin, glycyrrhizic acid, and chlorpromazine show efficacy in both in vitro and in silico environments. Chlorpromazine is the single agent that was found to have efficacy in in silico, in vitro, and in vivo areas.

In terms of mechanism of action, different targets from the structural and nonstructural proteins of COVID-19 were evaluated. Most of the studies focused on the main protease, papain-like protease, and spike glycoprotein. Apart from the specific protein that leads to viral replication, SARS-CoV-2 causes a surge of pro-inflammatory cytokines and chemokines, which cause damage to lung tissue and deterioration of lung function. Therefore, the design of a drug with multitarget of action against different proteins of COVID-19 and also anti-inflammatory potential could be valuable.

Currently, there is no highly efficacious and specific treatment for SARS-CoV-2. Herein, we provided data on novel compounds in therapeutic drug discovery and development. Due to the nature of SARS-CoV-2 and the rises of several high transmissible strains, repurposing existing drugs has demonstrated power by bringing several drugs to approval for treating COVID-19 patients, such as remdesivir. Our results confirmed that a large number of repurposed agents are currently being explored for treating SARS-CoV-2 infection. However, these drugs still suffer from suboptimal therapeutic effect or known strong side effect. To accelerate drug discovery and development, especially during the current pandemic, natural products capture attention again. Our results showed that various natural bioactive compounds are being investigated in the preclinical step of drug development for COVID-19. In addition to having high affinity, these herb active ingredients have antioxidant, vasoprotective, anticarcinogenic, and antiviral properties. Therefore, they can be used as extremely safe therapeutic compounds in drug design studies to control COVID-19. However, the pharmacological effects and adverse reactions of some drugs under development are still unclear, and hence, well-designed high-quality studies are needed to further study the effectiveness and safety of these potential drugs to accelerate drug development targeting SARS-CoV-2 and thus promote progress towards ending the pandemic.

## Figures and Tables

**Table 1:** Summary of studies with in silico method that used repurposed drugs for novel drug discovery against COVID-19.

Author	Year	Method	Detail of method	Name of compound/drug	Target	Efficacy	Comments
Abosheasha and El-Gowily [24]	2020	In silico	Drug repurposing molecular docking-based virtual screening	15 antiplatelet FDA-approved drugs	Main protease (Mpro) and spike glycoprotein (S)	Cilostazol has favorable binding interaction with Mpro (PDB ID: 6LU7) cilostazol, iloprost, epoprostenol, prasugrel, and icosapent ethyl that have a higher binding affinity on spike glycoprotein (S)	Cilostazol is a promising FDA drug against COVID-19 by inhibiting both Mpro and S protein
Abdul Kadhim et al. [25]	2020	In silico	Drug repurposing, docking	Experimental and approved drugs	Papain-like protease and RNA polymerase	Drugs that shared >70% similarity to the binding sites of those targets were reversin, pentagastrin, remdesivir, norfloxacin, and nitazoxanide against COVID-19 papain-like protease whereas benzylglutathione, lopinavir, and hydroxymethylglutathione against RNA polymerase	Antiresistance reversin showed the highest inhibitory efficacy against COVID-19 papain-like protease, and benzylglutathione is an experimental compound; however, it had the highest RNA polymerase inhibiting efficacy
Abu-Saleh et al. [26]	2020	In silico	Ligand-based/structure-based virtual screening, MD simulations, and binding energy calculations	Approved drugs and bioactive compounds listed in the DrugBank and ChEMBL databases	Main protease	Best MM/GBSA binding energy; ChEMBL275592, montelukast, ChEMBL28834. Bromocriptine and saquinavir demonstrate stability in the active site of Mpro	
Achilonu et al. [27]	2020	In silico	High-throughput virtual screening and ligand docking	FDA-approved drugs	Main protease	Isavuconazonium, a P2-P3 *α*-ketoamide derivative, and pentagastrina are the top three molecules (Lig13b as the benchmark) based on docking energy	
Aftab et al. [28]	2020	In silico	Repositioning/target-based virtual screening and molecular docking	Ten antiviral drugs were screened: ribavirin, remdesivir, sofosbuvir, penciclovir, nitazoxanide, nafamostat, chloroquine, galidesivir, favipiravir, and interferon	RNA-dependent RNA polymerase (RdRp)	Galidesivir and its drug-like compounds CID123624208 and CID11687749 have shown an effective attachment to the priming site of viral RdRp	CID123624208 and CID11687749 may be considered for in vitro and in vivo clinical trials
Ahmadi et al. [29]	2021	In silico	Drug repurposing study using molecular docking	Enfuvirtide, an HIV-1 fusion inhibitor peptide	SARS-CoV-2 fusion inhibitor	Enfuvirtide binding to the S2 protein of SARS-CoV-2 was remarkably stable and can act as a strong SARS-CoV-2 fusion inhibitor	
Ahmed et al. [30]	2021	In silico	Drug repurposing (high-throughput virtual screening) (HTVS) followed by re-docking with standard precision (SP) and extra-precision (XP) molecular docking	FDA-approved antiviral and anti-infection drugs	Main protease	Of 1397 potential drugs, 157 showed considerable affinity towards Mpro. High-affinity lead drugs (iodixanol, amikacin, troxerutin, and rutin) were identified. Amikacin was found to be the most potent inhibitor of main protease	Aminoglycosides may serve as a scaffold to design potent drug molecules against COVID-19
Anand et al. [31]	2021	In silico	Molecular docking	130 US FDA-approved drugs including hypertension, cardiovascular diseases, respiratory tract infections (RTI), antibiotics, and antiviral drugs	Structural and nonstructural proteins of SARS-CoV-2 (nsp3, nsp5, nsp10, nsp16)	15 potent drugs exhibiting significant inhibitory potential against SARS-CoV-2 like baloxavir marboxil, danoprevir and sofosbuvir, fosinopril, moexipril, quinapril, telmisartan, azilsartan, verapamil, and doxazosin	Azithromycin, doxycycline, clarithromycin, rifamycin, and augmentin: nsp10; virginiamycin, tunicamycin, quinupristin, fidaxomicin, digoxin, and azithromycin: main protease; caspofungin, amphotericin B, ketoconazole, and micafungin: E and N proteins; virginiamycin and amphotericin B: S protein
Ancy et al. [32]	2020	In silico	Molecular docking, molecular dynamics, and binding free energy simulation study	HIV-1 protease, namely, TMB607 and TMC-310911	Main protease	TMB607 molecule binds strongly with the SARS-CoV-2 main protease enzyme	
Ansari et al. [33]	2020	In silico	Repurposing drug molecular docking	TAT-peptide 47–57 (GRKKRRQRRRP)-conjugated repurposed drugs (i.e., lopinavir, ritonavir, favipiravir, and hydroxychloroquine)	Main protease	TP-conjugated ritonavir, lopinavir, favipiravir, and hydroxychloroquine have superior and significantly enhanced interactions with main protease	
Arun et al. [34]	2020	In silico	Repurposing drug molecular docking	Drugs available in the super DRUG2 database	Main protease	Binifibrate and bamifylline bind strongly to the enzyme active site	
Arya et al. [35]	2020	In silico	Molecular docking	FDA-approved drugs	Papain-like protease	15 FDA-approved drugs, including chloroquine and formoterol, bind the target enzyme with significant affinity and good geometry, suggesting their potential to be utilized against the virus	
Baby et al. [36]	2020	In silico	Schrodinger's computer-aided drug discovery tools for in silico drug repurposing	FDA-approved library of drugs	RNA-dependent RNA polymerase (RdRp)	Pitavastatin, ridogrel, rosoxacin	
Baby et al. [36]	2021	In silico	Schrodinger's computer-aided drug discovery tools for in silico drug repurposing	FDA-approved library of drugs	Main protease	Tipiracil and aprepitant interacted with the main protease	
Baker et al. [37]	2021	In silico	Repurposing drug molecular docking	50 compounds with activity against main protease	Main protease	Drugs including boceprevir, ciluprevir. narlaprevir, and telaprevir may be more potent against main protease than boceprevir and suitable for rapid repurposing	
Bharath et al. [38]	2020	In silico	Drug repurposing computer-aided drug design (CADD)	4015 known and approved small molecules	Spike glycoprotein	Glycyrrhizic acid (GA) of plant origin may be repurposed for SARS-CoV-2 intervention	
Bhowmik et al. [39]	2021	In silico	Repurposing drugs, docking, and molecular dynamic simulation	Orientin (phytochemical)	Inhibitor of SARS-CoV-2 spike and host cell receptor GRP78 binding	Binding of orientin in the overlapping residues of GRP78 binding region of SARS-CoV-2 spike model	As a promising precautionary or therapeutic measure for COVID-19
Bolelli et al. [40]	2021	In silico	Drug repurposing, virtual screening method	FDA-approved drugs	Main protease	Three compounds (dobutamine and its two derivatives)	
Cavasotto et al. [41]	2021	In silico	Drug repurposing, docking-based screening using a quantum mechanical scoring	FDA-approved drugs	Spike protein, main protease papain-like protease	Sovaprevir, elbasvir, danoprevir, samatasvir, Candesartan, saquinavir ritonavir, indinavir, lopinavir, brilacidin, flovagatran, aplidin, desmopressin, and felypressin listed as potential inhibitors of main protease	
Chandel et al. [42]	2020	In silico	Drug repurposing, molecular dynamics, and docking	FDA-approved drugs	Nsp9 replicase and spike proteins	Conivaptan exhibited the highest binding of the Nsp9 replicase. Tegobuvir exhibited maximum stability along with the highest binding energy at the active site of the spike proteins	
Chen et al. [43]	2020	In silico	Drug repurposing, molecular dynamics, and docking	FDA-approved drugs	Spike (S)-mediated cell entry	Cepharanthine, abemaciclib, osimertinib, trimipramine, colforsin, and ingenol	
Chidambaram et al. [44]	2020	In silico	Molecular docking	Coumarins and their analogs	Main protease	Natural coumarin analog toddacoumaquinone displayed remarkable inhibition ability. Synthetic coumarin analog (1 m) also displayed the comparable inhibition ability main protease in intricate with *α*-ketoamide	
Choudhary et al. [45]	2020	In silico	Drug repurposing, molecular dynamics, and docking	FDA-approved drugs	Spike glycoprotein and cellular angiotensin-converting enzyme 2 (ACE2) receptor	GR 127935 hydrochloride hydrate, GNF-5, RS504393, TNP, and eptifibatide acetate were found binding to virus binding motifs of ACE2 receptor. KT203, BMS195614, KT185, RS504393, and GSK1838705A were identified to bind at the receptor-binding site on the viral S protein	
Clemente et al. [46]	2021	In silico	Molecular docking, molecular dynamic (MD) simulations	Ibuprofen	Main protease	Racemic mixtures of the ibuprofen enantiomers might be a potential treatment for main protease	
Cosic et al. [47]	2021	In silico	Extended resonant recognition model (RRM)	Ivermectin	Spike proteins	Ivermectin could interfere with activity of spike proteins	
Daoud et al. [48]	2021	In silico	Structure-based pharmacophore approach, molecular docking, and repurposing studies	FDA-approved drugs	Main protease	Lopinavir, remdesivir, ritonavir, saquinavir, and raltegravir were successfully docked into the binding site of main protease	
de Oliveira et al. [49]	2021	In silico	Molecular modeling and virtual screening and repurposing studies	9091 FDA-approved drugs	Spike protein	24 best scored ligands (14 traditional herbal isolates and 10 approved drugs) as potential candidates to inhibit the S protein	Quinupristin, nilotinib, acetyldigitoxin
Delijewski and Haneczok [50]	2021	In silico	Supervised machine learning model and repurposing studies	FDA-approved drugs	Against SARS-CoV-2	Zafirlukast as the best repurposing candidate for COVID-19	
Dey et al. [51]	2021	In silico	Virtual database screening, molecular docking, all-atom molecular dynamic simulation, and MM-PBSA analysis	Tretinoin, mefenamic acid, ondansetron, and artemether	Envelope (E) protein	Tretinoin as a potential SARS-CoV-2 E protein ion channel blocker and virus assembly inhibitor	
Durdagi [52]	2020	In silico	Molecular modeling approach in virtual drug screening repurposing study	FDA-approved drugs	Type 2 transmembrane serine protease (TMPRSS2)	Benzquercin as strong TMPRSS2 inhibitor	
Durdagi et al. [53]	2020	In silico	Molecular docking, MM-GBSA-based predictions, and molecular dynamic repurposing study	FDA-approved drugs	Main protease and spike receptor-binding domain bound with ACE2 COVID-19 target proteins	Pimelautide, rotigaptide, telinavir, ritonavir, pinokalant, terlakiren, cefotiam, and cefpiramide as SARS-CoV-2 main protease inhibitors. Denopamine, bometolol, naminterol, rotigaptide, and benzquercin as potential ACE2/spike protein domain inhibitors	
Eleftheriou et al. [54]	2020	In silico	Molecular docking	34 approved and on-trial protease inhibitors	Main protease	HCV protease, DPP-4, *α*-thrombin, and coagulation factor Xa known inhibitors	
Elmezayen et al. [55]	2021	In silico	Molecular modeling approach in virtual drug screening repurposing study	Commercially available drugs and ZINC15 library	Main proteases	Four potential inhibitors against Mpro enzyme, two available drugs (Talampicillin and Lurasidone), and two novel drug-like compounds (ZINC000000702323 and ZINC000012481889)	
Encinar et al. [56]	2020	In silico	Molecular modeling approach in virtual drug screening and repurposing study	9000 US Food and Drug Administration (FDA)-approved investigational and experimental drugs from the DrugBank repository	S-Adenosyl-L-methionine-binding pocket of nsp16, [2] the unique “activating surface” between nsp16 and nsp10, and [3] the RNA-binding groove of nsp16	Tegobuvir, sonidegib, siramesine, antrafenine, bemcentinib, itacitinib, or phthalocyanine antagonism of SARS-CoV-2 RNAs lacking 20-O-methylation	
Farag et al. [57]	2020	In silico	Molecular modeling approach in virtual drug screening and repurposing study	2000 FDA-approved drugs	Main protease	Darunavir, nelfinavir, and saquinavir bound to the central site of main protease substrate-binding pocket rosuvastatin, montelukast, and the anti-histaminic fexofenadine bound to the terminal site of main protease substrate-binding pocket	Starting point for further in vitro and in vivo testing
Feng et al. [58]	2020	In silico	Molecular modeling approach in virtual drug screening and repurposing study	FDA-approved drugs	Spike protein	Eltrombopag possesses a high binding affinity to S protein plus human ACE2	
Ferraz et al. [59]	2020	In silico	Ligand and structure-based virtual screening, repurposing study	FDA-approved drugs	Main protease	Two oral (bedaquiline and glibenclamide) and one buccal drug (miconazole)	
Fischer et al. [60]	2020	In silico	Molecular docking approach in virtual drug screening and repurposing study	Over 606 million compounds	Main protease	12 purchasable compounds, with binding affinity to the target protease the natural compounds (−)—taxifolin and rhamnetin as potential inhibitors of main protease	
Gimeno et al. [61]	2020	In silico	Molecular modeling approach in virtual drug screening and repurposing study	FDA-approved drugs	Main protease	Perampanel, carprofen, celecoxib, alprazolam, trovafloxacin, sarafloxacin, and ethyl biscoumacetate. Carprofen and celecoxib	Initiative for in vitro testing
Guo et al. [62]	2020	In silico	Molecular modeling approach in virtual drug screening and repurposing study single-cell RNA sequencing	US FDA-approved drugs	Against SARS-CoV-2	281 FDA-approved drugs that have the potential to be effective against SARS-CoV-2 infection, 16 of which are currently undergoing clinical trials to evaluate their efficacy against COVID-19	Including the HIV protease inhibitor lopinavir/ritonavir combination (phase 4), glucocorticoid receptor agonist dexamethasone (phase 3/4), DNA replication inhibitor niclosamide (phase 2/3), antineoplastic agent lenalidomide (phase 4), and calcineurin inhibitor tacrolimus (phase 3), ABT-737 (BCL inhibitor), brefeldin-A (protein synthesis inhibitor), indirubin (CDK inhibitor), TPCA-1 (IKK inhibitor), lopinavir (HIV protease inhibitor), GW-441756 (growth factor receptor inhibitor), treprostinil (prostacyclin analog), tyrphostin-AG-1478 (EGFR inhibitor) and epoxycholesterol (LXR agonist), fostamatinib (SYK inhibitor), VER-155008 (HSP inhibitor), KU-0063794 (MTOR inhibitor), PIK-90 (PI3K inhibitor), linsitinib (IGF-1 inhibitor), TAK-715 (p38 MAPK inhibitor), Y-27632 (Rho-associated kinase inhibitor), AZ-628 (RAF inhibitor), and lestaurtinib (FLT3 inhibitor)
Gupta et al. [63]	2020	In silico	Molecular modeling approach in virtual drug screening and repurposing study	FDA-approved drugs	Main protease	Cobicistat is the most efficient inhibitor of Mpro both in silico and in vitro	
Huynh et al. [64]	2021	In silico	Docking and molecular dynamics and repurposing study	FDA-approved drugs	Papain-like protease	The chances of drug repurposing for PLpro might be low	
Ibrahim et al. [65]	2020	In silico	Molecular dynamic simulations, molecular docking, MM-GBSA analysis, and repurposing study	DrugBank database	Main protease	DB02388 and cobicistat (DB09065)	
Iftikhar et al. [66]	2020	In silico	Molecular modeling approach in virtual drug screening and repurposing study	4574 compounds also containing FDA-approved drugs	RdRp, main protease, and helicase	Rimantadine, bagrosin, and grazoprevir showed binding to main protease. Casopitant is a neurokinin-1 receptor that showed binding to RdRp. Meclonazepam and oxiphenisatin showed specific interactions with helicase	
Jain and Mujwar [67]	2020	In silico	Computational drug repurposing docking simulations	2880 FDA-approved drugs	Main protease	Metocurine, dihydroergotoxine, imatinib, daunorubicin, bromocriptine, irinotecan, azelastine, gestodene, adapalene, and simvastatin	Metocurine was chosen as a safe and effective drug candidate for developing therapy against the viral Mpro enzyme of SARS-CoV-2 for the treatment of COVID-19
Jarvis et al. [68]	2020	In silico	Tier-based scoring system repurposing study	Clinically developed drugs	Potential repurposing against COVID-19	Four drug classes (antimalarial amino-quinolones, selective estrogen receptor modulators (SERMs), low potency tricyclic antipsychotics, and tricyclic antidepressants) as potential drug candidates for COVID-19	The tricyclic antipsychotics and tricyclic antidepressants were further excluded based on a high adverse event profile
Kadioglu et al. [69]	2021	In silico	Repositioning/virtual drug screening, molecular docking, and supervised machine learning algorithm drug repositioning	FDA-approved drug natural compound dataset ZINC database	Spike protein, nucleocapsid protein, and 2′-o-ribose methyltransferase	Conivaptan, paritaprevir, simeprevir, dihydroergotamine, ZINC000027215482, ZINC000252515584, loniflavone, procyanidin	
Kandeel et al. [70]	2020	In silico	Drug repurposing molecular dynamic (MD) simulations followed by molecular mechanics/generalized born surface area (MM/GBSA) binding energy calculations	1697 clinical FDA-approved drugs	Papain-like protease	Phenformin, quercetin, and ritonavir	Phenformin was more stable than quercetin and ritonavir
Kandwal and Fayne [71]	2020	In silico	Repurposing drug computational design pharmacophore features	In-development/approved drugs	Viral nucleocapsid and nonstructural proteins	Isepamicin and streptomycin (nsp3); coenzyme-I, rutin, epigallocatechin gallate-(-), and procyanidin-b-2 (nsp7/nsp8/nsp12); paromomycin (nsp10/nsp16); olomoucine, sapropterin, tetrahydrofolic acid, INS316, and adenosine phosphate (nsp15); varespladib, hexanoic acid, citric acid, OSI-027, MK-5108, stepronin, calcium gluceptate, CPP, pirenoxine, midafotel, and maltobionic acid (nucleocapsid)	
Khan et al. [72]	2020	In silico	Drugs repurposing molecular dynamic simulation	31 FDA-approved anti-HIV drugs, and traditional Chinese medicines (TCM) database	Main protease	Saquinavir and TCM5280805	
Khan et al. [73]	2020	In silico	Drugs repurposing molecular docking	23 prospective drug candidates	Main protease	Epirubicin, vapreotide, and saquinavir exhibited better binding affinity	Synergistic interaction
Kouznetsova et al. [74]	2020	In silico	Drugs repurposing molecular docking	FDA-approved drugs	Papain-like protease	Inhibitors of HIV, hepatitis C, and cytomegalovirus (CMV) demonstrated some activity	
Krishnaprasad et al. [75]	2020	In silico	Drugs repurposing molecular docking	FDA-approved library of drugs	RNA-dependent RNA polymerase	Pitavastatin, ridogrel, and rosoxacin displayed superior binding with the active site	
Kumar et al. [76]	2020	In silico	Drugs repurposing docking and molecular dynamic (MD) simulations combined with molecular mechanics/generalized born surface area (MM/GBSA)	12 FDA-approved drugs (darunavir, indinavir, saquinavir, tipranavir, diosmin, hesperidin, rutin, raltegravir, velpatasvir, ledipasvir, rosuvastatin, and bortezomib)	Main protease	Saquinavir as a potent inhibitor of dimeric main protease	
Kumar et al. [77]	2020	In silico	Drugs repurposing molecular docking molecular dynamic simulations MM/GBSA	Withaferin A (Wi-A), withanone (Wi-N) (active withanolides of ashwagandha), and caffeic acid phenethyl ester (CAPE, bioactive ingredient of propolis)	Main protease	Wi-N and CAPE possess the potential to inhibit the functional activity of main protease	
Kumar et al. [78]	2020	In silico	Drugs repurposing molecular docking	FDA-approved drugs	Main protease	Lopinavir-ritonavir, tipranavir, and raltegravir show the best molecular interaction with the main protease	
Kumar et al. [79]	2020	In silico	Drugs repurposing molecular docking molecular dynamic simulations	FDA-approved library of drugs	Main protease	Hyaluronic acid and acarbose show strong interactions with catalytic site residues of main protease	
Li et al. [80]	2020	In silico	Drug repurposing free energy perturbation-based absolute binding free energy (FEP-ABFE) predictions	Virtual screening of existing drugs	Main protease	25 drugs were predicted, and 15 were confirmed as potent inhibitors of SARS-CoV-2 main protease. The most potent one is dipyridamole. Hydroxychloroquine (ki = 0.36 *μ*M) and chloroquine (ki = 0.56 *μ*M) were also found to potently inhibit main protease	
Liang et al. [81]	2021	In silico	Drug repurposing molecular docking	2,631 FDA-approved small molecules	Multiple main proteins	29 drugs that could actively interact with two or more target proteins, with 5 drugs (avapritinib, bictegravir, ziprasidone, capmatinib, and pexidartinib) being common candidates for all four key host proteins and 3 of them possessing the desirable molecular properties	
Lokhande et al. [82]	2021	In silico	Drugs repurposing molecular docking molecular dynamic simulations	FDA-approved drugs	Main protease	Mitoxantrone, leucovorin, birinapant, and dynasore	
Mahanta et al. [83]	2020	In silico	Drugs repurposing molecular docking molecular dynamic simulations	U.S. Food and Drug Administration-approved antimicrobial drugs	Main protease	Viomycin	
Mahdian et al. [84]	2021	In silico	Drugs repurposing molecular docking molecular dynamic simulations	FDA-approved drugs	Viral entry receptors (ACE2 and CD147) and integral enzyme of the viral polymerase (RdRp)	Ledipasvir, estradiol benzoate, and vancomycin and paritaprevir	
Marak et al. [85]	2020	In silico	Repurposing drug homology modeling molecular docking	108 FDA-approved antiparasitic and anti-inflammatory drugs	10 SARS-CoV-2 targets (PLpro, 3CLpro, RdRp, spike, helicase, NSP1, NSP3, NSP4, NSP9, and NSP16-NSP10)	Ivermectin, atovaquone, posaconazole, doxycycline, moxidectin, amphotericin B, chlortetracycline, spiramycin, sulfasalazine, parecoxib, and etoricoxib exhibited good binding affinities	
Mohapatra et al. [86]	2020	In silico	Repurposing drug machine learning (ML) technology	FDA-approved drugs	Against COVID-19	10 FDA-approved commercial drugs that can be used for repurposing amprenavir would probably be the most effective drug based on the selected criteria	
Molavi et al. [87]	2021	In silico	Repurposing drug molecular docking	1760 FDA-approved drugs	RNA-dependent RNA polymerase (RdRp) and main protease	Nilotinib, imatinib, and dihydroergotamine for 3clpro and dexasone and raltegravir for RdRp. Raltegravir, an anti-HIV drug, was observed to be the best compound against RdRp based on docking binding energy dihydroergotamine is a suitable candidate for main protease	
Mulgaonkar et al. [88]	2020	In silico/in vitro	Repurposing drug molecular docking	FDA-approved drugs	Spike glycoprotein	BCR-ABL tyrosine kinase inhibitor, imatinib, inhibits SARS-CoV-2	Via fusion inhibition
Mycroft-West et al. [89]	2020	In silico	Repurposing drug molecular docking molecular dynamic simulations	Heparin	Spike (S1) protein receptor-binding domain	Inhibition of viral infection arises from an overlap between the binding sites of heparin/HS on S1-RBD	Repurposing heparin and its derivatives as antiviral agents against SARS-CoV-2
Nayarisseri et al. [90]	2020	In silico	Shape-based machine learning assisted by molecular docking and molecular dynamic simulations. ADMET studies	31 repurposed compounds	Main protease	Remdesivir, valrubicin, aprepitant, and fulvestrant	The novel compound nCorv-EMBS herein proposed stands as a promising inhibitor to be evaluated further for COVID-19 treatment
Odhar et al. [91]	2020	In silico	Molecular docking molecular dynamic simulations	1615 FDA-approved drugs	Main protease	Conivaptan azelastine	
Ortega et al. [92]	2020	In silico	Repurposing drug molecular docking	Famotidine	Against SARS-CoV2	Famotidine could interact within the catalytic site of the three proteases associated with SARS-CoV2 replication	Weak binding affinity could be reached only upon intravenous administration
Pandey et al. [93]	2021	In silico	Repurposing drug molecular docking	9 flavonoids	Spike glycoprotein	Baicalin	
Parveen and Alnoman [94]	2021	In silico	Molecular docking molecular dynamic simulation density functional theory (DFT) ADME-Tox	FDA-approved anticancer drugs (capmatinib, pemigatinib, selpercatinib, and tucatinib)	Spike glycoprotein (S1) and the main protease	Potential of selected anticancer drugs for plausible drug development to fight COVID-19	Capmatinib, pemigatinib, selpercatinib, and tucatinib
Peele et al. [95]	2020	In silico	Molecular docking molecular dynamic simulations	USFDA-approved drugs, plant-derived natural drugs	Main protease	Lopinavir, amodiaquine, theaflavin digallate	
Pinzi et al. [96]	2021	In silico	Drug repurposing molecular docking molecular mechanic Poisson–Boltzmann surface area (MM-PBSA)	DrugBank database	Main protease	22 candidates with putative SARS-CoV-2 Mpro inhibitory activity. Enalkiren, ethylsulfonamide-D-Trp-Gln-p-aminobenzamidine, delparantag ritonavir and lopinavir, saquinavir	Beneficial polypharmacological effects
Pokhrel et al. [97]	2020	In silico	Drug repurposing molecular dynamic simulations	US Food and Drug Administration (FDA)-approved drugs	RNA-dependent RNA polymerase	Quinupristin is particularly interesting because it is expected to bind across the RNA tunnel, blocking access from both sides	Quinupristin represents a potential anti-SARS-CoV-2 therapeutic
Ray et al. [98]	2020	In silico	Drug repurposing intramolecularly quenched fluorescence (IQF) peptide substrate	774 FDA-approved drugs	Main protease	Ethacrynic acid, naproxen, allopurinol, butenafine hydrochloride, raloxifene hydrochloride, tranylcypromine hydrochloride, saquinavir mesylate	
Sachdeva et al. [99]	2020	In silico	Drug repurposing molecular docking	Antimalarial drugs	Spike protein and main protease	Doxycycline showed the most effective binding to the spike protein, whereas halofantrine and mefloquine bound effectively with the main protease	Doxycycline could potentially be a good candidate for repurposing for COVID-19
Sang et al. [100]	2020	In silico	Drug repurposing molecular docking molecular mechanics Poisson–Boltzmann surface area (MM-PBSA)	6 approved anti-HIV drugs	Main protease	Darunavir	
Saxena et al. [101]	2021	In silico/In vitro	Drug repurposing molecular docking	FDA-approved DrugBank database	Spike protein	Ertugliflozin possesses several desired properties	Good candidate for immediate repurposing for the treatment of COVID-19
Setianingsih et al. [102]	2020	In silico	Drug repurposing molecular docking, molecular dynamic simulations	160 potential drugs from therapeutic target database	13 protein targets (12 SARS-CoV-2 proteins and 1 human protein)	Suramin, the strongest binding affinity against 3 protein targets (spike protein, nucleocapsid protein, ACE2)	Suramin is the most potential to bind nucleocapsid and spike protein of SARS-CoV-2
Shah et al. [103]	2020	In silico	Drug repurposing molecular docking	61 molecules that are already being used in clinics or under clinical scrutiny as antiviral agents	Against the SARS-CoV-2	37 molecules were found to interact with >2 protein structures of COVID-19. HIV protease inhibitors and RNA-dependent RNA polymerase inhibitors showed promising features of binding to COVID-19 enzyme	Methisazone, an inhibitor of protein synthesis; CGP42112A, an angiotensin AT2 receptor agonist; and ABT450, an inhibitor of the nonstructural protein 3-4A, might become convenient treatment option as well against COVID-19
Sharma and Mishra [104]	2020	In silico	Drug repurposing target-based virtual ligand screening	ZINC drug database and our own database of natural products	Against the SARS-CoV-2	Antivirus drugs (ribavirin, valganciclovir, and thymidine), antibacterial drugs (cefpiramide, sulfasalazine, phenethicillin, lymecycline, demeclocycline, doxycycline, oxytetracycline, and tigecycline), anti-asthmatic drugs (montelukast, fenoterol, and reproterol), and hepatoprotective drug silybin have antiviral activity. Natural hesperidin was targeting the binding between spike RBD and human ACE2	The natural products, such as flavonoids like neohesperidin, hesperidin, baicalin, kaempferol 3-*O*-rutinoside, and rutin from different sources, andrographolide, neoandrographolide, and 14-deoxy-11,12-didehydroandrographolide from *A. paniculata*, and a series of xanthones from the plants of *Swertia* genus, with antivirus, antibacteria, and anti-inflammation activity could effectively interact with these targets of SARS-CoV-2
Shekhar et al. [105]	2020	In silico	Drug repurposing molecular docking molecular dynamic simulations	2,625 FDA-approved small molecules	Spike (S) protein fusion peptide region	Chloramphenicol succinate, imipenem, and imidurea	
Singh et al. [106]	2021	In silico	Drug repurposing molecular docking molecular dynamic simulations	1749 FDA-approved drugs	NSP12, a RNA polymerase	5 compounds which include 3a (paritaprevir), 3d (glecaprevir), 3h (velpatasvir), 3j (remdesivir), and 3l (ribavirin) had the best binding affinity	
Sinha et al. [107]	2021	In silico	Drug repurposing systematic pharmacokinetics, drug-likeness, basicity predictions, virtual screening, and molecular dynamic analysis	Hydroxychloroquine (HCQ), chloroquine (CQ)	Spike protein	1-[1-(6-Chloroquinolin-4-yl) piperidin-4-yl]piperidin-3-ol and (1r,2R)-2-N-(7-chloroquinolin-4-yl)cyclohexane-1,2-diamine interact with the active site of the spike protein similar to HCQ and CQ, respectively, with augmented safety profile	
Soni et al. [108]	2020	In silico	Molecular docking molecular dynamic simulation ADME properties	Rifampicin	Main protease	Rifampicin docking score was −7.24 kcal·mol^–1^, and it can predict as a very good inhibitor of main protease	
Tariq et al. [109]	2020	In silico	Drug repurposing molecular docking molecular dynamic simulations	15 antimalarial drugs (including chloroquine) and 2413 US Food and Drug Administration-approved drugs	Main protease spike (S) protein	Paromomycin with activity against two targets spike protein and protease domain	
Tatar et al. [110]	2021	In silico	Drug repurposing molecular docking molecular dynamic simulations	34 antiviral compounds	RNA-binding domain	Rapamycin, saracatinib, camostat, trametinib, and nafamostat were the top hit compounds	
Tejera et al. [111]	2020	In silico	Drug repurposing quantitative structure-activity relationship (QSAR) mode molecular docking molecular dynamic simulation MM-PBSA method	DrugBank database	Main protease	Levothyroxine, amobarbital, and ABP-700	
Teralı et al. [112]	2020	In silico	Drug repurposing molecular docking	7,173 clinically approved drug	Angiotensin-converting enzyme 2 (ACE2)	Lividomycin, burixafor, quisinostat, fluprofylline, pemetrexed, spirofylline, edotecarin, diniprofylline	
Trezza et al. [113]	2020	In silico	Drug repurposing docking simulations, with molecular dynamics (MD), supervised MD (SuMD), and steered MD (SMD) simulations	FDA-approved drugs	Spike glycoprotein	Simeprevir, lumacaftor	
Ugurel et al. [114]	2020	In silico	Drug repurposing structure-based drug design genome sequences were analyzed	FDA-approved drugs	Helicase (Nsp13)	Cangrelor, fludarabine, folic acid, and polydatin inhibit both the wild-type and mutant SARS-CoV-2 helicase	
Unni et al. [115]	2020	In silico	Drug repurposing molecular docking molecular dynamic simulations	DrugBank and PubChem library	Spike protein (S protein)	Bisoxatin (DB09219)	A laxative drug
Vaishali et al. [116]	2020	In silico	Drug repurposing molecular docking molecular dynamic simulation ADME properties	FDA-approved compounds	Nonstructural protein 9 (Nsp9) replicase and spike proteins	Conivaptan exhibited the highest binding energy and maximum stability of the Nsp9 replicase. Tegobuvir exhibited maximum stability along with the highest binding energy at the active site of the spike proteins	
Verma et al. [117]	2020	In silico	Drug repurposing molecular docking molecular dynamic simulation MM-GBSA-based energy	FDA-approved drugs	Main protease	Top-ranked drugs including adefovir, lumefantrine, dipyridamole, dihydroergotamine, hexoprenaline, riboflavin (vitamin B2), and pantethine (vitamin B5)	
Wei et al. [118]	2020	In silico	Drug repurposing molecular docking molecular dynamic simulations	US Food and Drug Administration (FDA)-approved drugs from DrugBank and natural compounds from traditional Chinese medicine systems pharmacology (TCMSP)	Spike protein (S protein)	Digitoxin and bisindigotin in TCMSP had the highest docking scores Forsythiae fructus and Isatidis radix are components of Lianhua Qingwen, and raltegravir had relatively high binding scores	
Xu et al. [119]	2021	In silico	Drug repurposing molecular docking molecular dynamic simulations	FDA-approved drugs	Spike protein	Thymoquinone, a phytochemical compound obtained from the plant Nigella sativa, is a potential drug candidate	

**Table 2:** Summary of studies with in silico method that used natural products for novel drug discovery against COVID-19.

Author	Year	Method	Detail of method	Name of compound/drug	Target	Efficacy	Comments
Abdelli et al. [120]	2021	In silico	Molecular docking	Isothymol, thymol, limonene, P-cymene, and *γ*-terpinene derived from the essential oil of the antiviral and antimicrobial plant Ammoides verticillata (Desf.) Briq.	Inhibition of ACE2 cellular receptor	Isothymol, a major component of this plant, gives the best docking scores, as good ACE2 inhibitor	
Abouelela et al. [121]	2021	In silico	Molecular docking, dynamic simulation, and binding free energy calculation	Aloe	Main protease and spike protein	132, 134, and 159 were the best scoring compounds against main protease, while compounds 115, 120, and 131 were the best scoring ones against spike glycoprotein. Compounds 120 and 131 were able to achieve significant stability and binding free energies during molecular dynamic simulation	
Adem et al. [122]	2020	In silico	Molecular docking	Medicinal plant-based bioactive compounds (80 flavonoid compounds)	Main protease	Hesperidin, rutin, diosmin, apiin, diacetylcurcumin, (E)-1-(2-hydroxy-4-methoxyphenyl)-3-[3-[(E)-3-(2-hydroxy-4-methoxyphenyl)-3-oxoprop-1-enyl]phenyl]prop-2-en-1-one, and beta, beta′-(4-methoxy-1,3-phenylene)bis(2′-hydroxy-4′,6′-dimethoxyacrylophenone have been found as more effective on COVID-19 than nelfinavir	
Allam et al. [123]	2020	In silico	Molecular docking, 3D shape similarity study (rapid overlay chemical similarity-ROCS) to the clinically used drugs in COVID-19 patients	3′-Hydroxy-4′-methoxy-chroman-7-O-*β*-d-glucopyranoside 4, ferulic acid heptyl ester 1, naringenin 2, and 4,2′,4′-trihydroxy-6′-methoxychalcone-4′-O-*β*-d-glucopyranoside 3, which were isolated from peach (Prunus persica (L.) Batsch) fruits	Main protease, spike protein	Naringenin 2 and 4,2′,4′-trihydroxy-6′-methoxychalcone-4′-O-*β*-d-glucopyranoside 3 have a strong binding mode to a protease receptor and spike protein and also block the inflammatory storm	Recommendation of peach fruits in controlling and managing COVID-19 cases
Al‐Sehemi et al. [124]	2020	In silico	Molecular docking	31000 natural compounds of the natural product activity and species source (NPASS) library	Spike glycoprotein	Castanospermine and karuquinone B were shown to be the best-in-class derivatives in silico able to target an essential structure of the virus and to act in the early stage of infection	
Attia et al. [125]	2021	In silico	Molecular docking	10 phenolic antiviral	Against SARS-CoV-2	Hesperidin showed the highest docking score	Hesperidin and its mediated ZnO nanoparticles are willing antiviral agents
Azim et al. [126]	2020	In silico	Virtual screening methods molecular docking	27 plant metabolites	Main protease proteins (MPP), Nsp9 RNA-binding protein, spike receptor-binding domain, spike ectodomain, and HR2 domain	Asiatic acid, avicularin, guajaverin, and withaferin showed a maximum binding affinity with all key proteins in terms of lowest global binding energy	
Bhowmik et al. [39]	2021	In silico	Repurposing drugs, docking, and molecular dynamic simulation	Orientin (phytochemical)	Inhibitor of SARS-CoV-2 spike and host cell receptor GRP78 binding	Binding of orientin in the overlapping residues of GRP78 binding region of SARS-CoV-2 spike model	As a promising precautionary or therapeutic measure for COVID-19
Çakır et al. [127]	2021	In silico	Molecular docking	Peptides derived from beta-lactoglobulin	Inhibit the host cell membrane receptors	Ala-Leu-Pro-Met-His-Ile-Arg (ALMPHIR) and Ile-Pro-Ala-Val-Phe-Lys (IPAVFK) peptides	*β*-Lactoglobulin (BLG) is the major whey protein of cow and sheep's milk (∼3 g/l)
Chatterjee et al. [128]	2021	In silico	Molecular docking	Hesperidin, kaempferol, quercetin, epigallocatechin	PLpro (papain-like protease), RdRp (RNA-dependent RNA polymerase), Mpro or 3cl protease, and spike protein	Hesperidin, kaempferol, quercetin, epigallocatechin	Lead to conclusive data for the treatment of polyphenols, flavonoids, and bioflavonoids against SARS-CoV-2
Chikhale et al. [129]	2020	In silico	Molecular docking, dynamics	Plant Withania somnifera (Indian ginseng)	NSP15 endoribonuclease and receptor-binding domain of prefusion spike protein	Withanoside X and quercetin glucoside from W. somnifera have favorable interactions at the binding site of selected proteins	Immunomodulatory, antioxidant, and anti-inflammatory roles
Chikhale et al. [130]	2021	In silico	Molecular docking, dynamics, and network pharmacology analysis	Saikosaponins	Adjuvant therapy in the treatment of COVID-19	Saikosaponins interact with the proteins CAT gene CAT (catalase) and checkpoint kinase 1 (CHEK1)	Possible improvement in immune response towards COVID-19
Chikhalet al. [131]	2020	In silico	Molecular docking, dynamics	Asparagus racemosus (Willd.)	NSP15 endoribonuclease and spike receptor-binding domain	Asparoside -C and Asparoside -F have good binding with target proteins	Asparagus racemosus holds promise as SARS-CoV-2 (S) and (N) protein inhibitor
Chowdhury [132]	2020	In silico	Molecular docking, dynamics	Tinospora cordifolia (Giloy)	Main protease	Berberine can regulate main protease protein's function	
Dahab et al. [133]	2020	In silico	Molecular docking	10 phenolic compounds of different classes (phenolic acids, flavonoids, and coumarins)	Main protease and RNA polymerase	The top 7 hits are flacourticin [3], sagerinic acid [16], hordatine a [23], hordatine B [134], N-feruloyl tyramine dimer [135], bisavenanthramides B-5 [27], and vulnibactins [38] and have better binding scores than remdesivir, the native ligand in RNA polymerase target (PDB ID: 7bV2)	Hordatines are phenolic compounds present in barley and were found to exhibit the highest binding affinity to both protease and polymerase
Das et al. [136]	2020	In silico	Molecular docking	Flavonoid-based phytochemicals of Calendula officinalis	Main protease	Rutin, isorhamnetin-3-O-*β*-D, calendoflaside, narcissin, calendulaglycoside B, calenduloside, and calendoflavoside have better binding energy than the native ligand	Rutin and caledoflaside showed better stability, compactness, and flexibility
Debnath et al. [137]	2020	In silico	Sequential E-pharmacophore and structure-based virtual screening (VS)	113687 number of commercially available natural compounds	ADP-ribose phosphatase	6 potential inhibitors having good binding affinity towards active sites	Commercially available
Dev and Kaur [138]	2020	In silico	Molecular docking	Eucalyptus essential oil	Main protease	Jensenone may represent potential treatment potential to act as main protease inhibitor	
Duru et al. [139]	2021	In silico	Molecular docking	Oil of Nigella sativa seed	Replicase polyprotein 1a, RNA-binding protein of NSP9, ADP ribose phosphatase of NSP3, 3-chymotrypsin-like protease 3CLpro, and RNA-dependent RNA polymerase RdRp, and ACE2-angiotensin-converting enzyme from the Homo sapiens	The binding affinity of caryophyllene oxide was the highest on NSP9 and RdRp targets, while *α*-bergamotene gave the best binding affinity on RPIA target. The binding affinity of *β*-bisabolene on the ACE2 was almost the same as remdesivir	
El-Demerdash et al. [140]	2021	In silico	Molecular dynamic simulations, molecular docking	15 guanidine alkaloids	Main protease (Mpro) (PDB ID: 6lu7), spike glycoprotein (PDB ID: 6VYB), nucleocapsid phosphoprotein (PDB ID: 6VYO), membrane glycoprotein (PDB ID: 6M17), and a nonstructural protein (nsp10) (PDB ID: 6W4H)	Crambescidin 786 [5] and crambescidin 826 had the highest binding affinities. The examined 15 alkaloids especially 5 and 13 showed promising docking, ADMET, toxicity, and MD results	
Elekofehinti et al. [141]	2020	In silico	Molecular docking studies, molecular dynamics, and ADME/Tox	50,000 natural compounds retrieved from IBS database	Papain-like protease	STOCK1N-69160 [(S)-2-((R)-4-((R)-2-amino-3-methylbutanamido)-3-(4-chlorophenyl) butanamido)propanoic acid hydrochloride] has been proposed as a novel inhibitor against COVID-19 PLpro	
El‐Hawary et al. [142]	2021	In silico	Molecular docking (a combination of metabolomics and in silico approaches)	A. terreus, the endophytic fungus associated with soybean roots	Main protease	Aspergillide B1 and 3*α*-hydroxy-3,5-dihydromonacolin L were found to be potent anti-COVID-19 drug candidates	
Emirik [143]	2020	In silico	Molecular docking, MM-GBSA-based predictions, and molecular dynamics	Turmeric contents	SARS-CoV-2 vital proteins	Turmeric spice has the potential to inhibit the SARS-CoV-2 vital proteins and can be used a therapeutic or protective agent against SARS-CoV-2 via inhibiting key protein of the SARS-CoV-2. Compounds 4, 23, and 6 are the most prominent inhibitor for the main protease, the spike glycoprotein, and RNA polymerase of virus, respectively	
Fakhar et al. [144]	2020	In silico	Structure-based pharmacophore modeling, virtual screening-based PHASE screen score, molecular modeling	Anthocyanin derivatives	Main protease	6 best anthocyanin-derived natural compounds, which could be used as promising lead compounds against main protease SARS-CoV-2	
Falade et al. [145]	2021	In silico	Molecular docking	Saponins and tannins	Main protease	Ellagic acid, arjunic acid, theasapogenol B, and euscaphic acid as potential inhibitors of SARS-CoV-2 (Mpro) with better pharmacokinetics and bioavailability compared with remdesivir	
Fitriani et al. [146]	2020	In silico	Molecular docking	Phytochemical compounds (Moringa oleifera, Allium cepa, Cocos nucifera, Psidium guajava, and Eucalyptus globulus)	Main protease	Oleanolic acid in Allium cepa, *α*-tocotrienol in Cocos nucifera, asiatic acid in Psidium guajava, and culinoside in Eucalyptus globulus were the most recommended compound in each medicinal plant	Oleanolic acid in Allium cepa found as a potential inhibitor of COVID-19 Mpro
Gangadevi et al. [147]	2021	In silico	Molecular dynamic simulations, molecular docking	Library of natural compounds	Host ACE2 receptor with spike RBD domain of SARS-CoV-2	Kobophenol A, identified through docking studies, is the first compound that inhibits SARS-CoV-2 binding to cells through blocking S1-RBD to the host ACE2 receptor and thus may serve as a good lead compound against COVID-19	
Gangarapu et al. [148]	2020	In silico	Molecular docking online pkCSM and SwissADME Web server	Phytoconstituents of Siddha official formulation kabasura kudineer and novel herbal preparation—JACOM	Spike protein	37 compounds were screened, and of these, 9 compounds showed high binding affinity against spike protein	SNACK-V formulations could be used for effective treatment of COVID-19
Ghosh et al. [149]	2020	In silico	Molecular dynamic simulations, molecular docking	8 polyphenols from green tea	Main protease	3 polyphenols (epigallocatechin gallate, epicatechin gallate, and gallocatechin-3-gallate) interact strongly with one or both catalytic residues (His41 and Cys145) of main protease	
Ghosh et al. [150]	2021	In silico	Molecular dynamic simulations, molecular docking, MM-GBSA analysis	Justicia adhatoda alkaloids	Main protease	1 alkaloid (anisotine) had interaction with both the catalytic residues (His41 and Cys145) of Mpro and exhibited good binding affinity (−7.9 kcal/mol)	More potent Mpro inhibitor than the two previously recommended antiviral drugs (lopinavir and darunavir)
Gorla et al. [151]	2020	In silico	Molecular docking	Essential flavonoids	SARS-CoV-2 spike glycoprotein receptor-binding domain (RBD-S) and host angiotensin-converting enzyme-2 protease domain (PD-ACE2)	Biochanin A and silymarin bind significantly at the active sites of RBD-Sand PD-ACE2	
Gurung et al. [152]	2020	In silico	Virtual screening, molecular docking	Antiviral compounds from plants	Main protease	Bonducellpin D was identified as the best lead molecule, which shows higher binding affinity	
Gyebi et al. [153]	2021	In silico	Molecular docking, ADME/Tox, and Lipinski filter analysis	African plants derived alkaloids and terpenoids	Main protease	4 nontoxic, druggable plant-derived alkaloids (10-hydroxyusambarensine and cryptoquindoline) and terpenoids (6-oxoisoiguesterin and 22-hydroxyhopan-3-one)	
Elwakil et al. [154]	2021	In silico	Gas chromatography/mass spectrometry analysis molecular docking	Egyptian propolis	RNA-dependent RNA polymerase, spike protein S1, and main protease	Octatriacontyl pentafluoropropionate is well oriented inside the enzyme pockets, in addition to an excellent binding manner with the active site of the target macromolecules	Menoufia propolis could be a promising candidate in the combat against the pandemic COVID-19
Hasan et al. [155]	2020	In silico	Molecular docking	Compounds present in the plant Solanum surattense	Main protease	13 phytochemicals were studied, eight showed very strong binding affinities to main protease, and four showed moderate to strong binding affinities	
Hashem [156]	2020	In silico	Molecular docking	Honeybee and propolis	Main protease	6 main compounds possess high binding energy with the receptor active site of the main protease	
Ibrahim et al. [157]	2020	In silico	Molecular dynamic simulations, molecular docking, MM-GBSA analysis	MolPort database that contains over 100,000 natural products	Main protease	9 potent natural products with binding affinities (ΔG binding) >−48.0 kcal/mol four bis([1, 3]dioxolo)pyran-5-carboxamide derivatives were identified as potential drug candidates	MolPort-004-849-765, MolPort-000-708-794, MolPort-002-513-915 and MolPort-000-702-646 are bis([1,3]dioxolo)pyran-5-carboxamide derivatives
Ibrahim et al. [158]	2020	In silico	Molecular dynamic simulations, molecular docking	Metabolites present in several common spices	Main protease	High potency of salvianolic acid A and curcumin as main protease inhibitors	Salvianolic acid A as an in silico natural product inhibitor against the SARS-CoV-2 main protease
Isa et al. [159]	2020	In silico	Docking and molecular dynamic (MD) simulation	Extracts of Zingiber officinale and Anacardium occidentale	Main protease	Six compounds had good binding energies. CID_9910474 and CID_10503282 had a better stability when compared to other selected phytochemicals	
Istifli et al. [160]	2020	In silico	Molecular dynamics and molecular mechanic Poisson–Boltzmann surface area (MM/PBSA) methods	23 phytochemicals belonging to different flavonoid subgroups	Spike glycoprotein cellular proteases (transmembrane serine protease 2 (TMPRSS2), cathepsin B and L (CatB/L)).	(−)-Epicatechin gallate interacted strongly with all the proteins studied	Epicatechin gallate can be evaluated as a candidate molecule in drug development studies against 2019-nCoV since it was not the substrate of P-gp (P-glycoprotein), did not inhibit any of the cytochrome Ps, and did not show AMES toxicity or hepatotoxicity on eukaryotic cells
Jan et al. [161]	2021	In silico	Cell-based infection assay molecular modeling	2,855 small molecules and 190 traditional herbal medicines	Main protease RNA-dependent RNA polymerase	Mefloquine, nelfinavir, and extracts of Ganoderma lucidum (RF3), Perilla frutescens, Mentha haplocalyx	
Jo et al. [162]	2020	In silico	Docking	Flavonoids	Main protease	Baicalin, herbacetin, and pectolinarin have been discovered to block the proteolytic activity. Baicalin showed an effective inhibitory activity against main protease	
Joshi et al. [163]	2021	In silico	Docking	7100 molecules	Main protease	Several natural molecules such as *δ*-viniferin, myricitrin, taiwanhomoflavone A, lactucopicrin 15-oxalate, nympholide A, afzelin, biorobin, hesperidin, and phyllaemblicin B strongly binds to main protease	These molecules also showed strong binding with other potential targets of SARS-CoV-2 infection such as viral receptor human angiotensin-converting enzyme 2 (hACE2) and RNA-dependent RNA polymerase (RdRp)
Junior et al. [164]	2021	In silico	Docking and molecular dynamic (MD) simulation	Lapachol(1,4-naphthoquinone)	SARS-CoV-2 nonstructural proteins (nsps)	Lapachol derivatives VI and IX demonstrated the strongest binding	Lapachol derivatives are potential ligands for SARS-CoV-2 Nsp9
Kar et al. [165]	2020	In silico	Molecular docking molecular dynamic simulations and analysis of MM-PBSA energy	Indian plants including Justicia adhatoda, Ocimum sanctum, and Swertia chirata	Spike protein, main protease enzyme Mpro, and RNA-dependent RNA polymerase (RdRp)	Anisotine against SARS-CoV-2 spike and Mpro proteins and amarogentin against RdRp as potential inhibitors	
Khalifa et al. [166]	2020	In silico	Molecular docking modeling structural-relationship activity	10 anthocyanins	Main protease	Phacelianin, gentiodelphin, cyanodelphin, tecophilin	Leading molecules for further optimization and drug development process to combat COVID-19
Khalifa et al. [167]	2020	In silico	Molecular operating environment molecular docking	19 structural different hydrolysable tannins	Main protease	Pedunculagin, tercatain, and castalin	
Khan et al. [168]	2020	In silico	Molecular docking	Marine natural products	Main protease	C-1 (PubChem CID 11170714) exhibited good activity	
Kiran Raj et al. [169]	2020	In silico	Molecular docking	C-Phycocyanin of Spirulina platensis	Nonstructural proteins 12	C-Phycocyanin inhibits the active site of nsp12	
Krupanidhi et al. [170]	2020	In silico	Molecular docking molecular dynamic simulation ADME along with toxicity analysis	Phytochemical constituents of Tinospora cordifolia	Main protease	Tinosponone	
Kumar et al. [171]	2020	In silico	Molecular docking molecular dynamic simulations and analysis of MM-PBSA energy	Novel natural metabolites	Main protease	Ursolic acid, carvacrol, and oleanolic acid could	
Kumar et al. [172]	2021	In silico	Molecular docking, ADMET, and molecular dynamic simulations	Phytoconstituents from natural herbs	Main protease	Laurolitsine	Laurolitsine, an active constituent of roots of Lindera aggregata
Kumar et al. [173]	2021	In silico	Molecular docking molecular dynamic simulations and analysis of MM-PBSA energy	Strychnos nux-vomica	Main protease	Demethoxyguiaflavine, strychnoflavine	
Li et al. [174]	2021	In silico	Network pharmacology and in vitro experiment verification molecular docking	Maxing Shigan decoction (MXSGD)	ACE2, Mpro, and RdRp	The components with strong potential affinity (top 10) with ACE2, Mpro, and RdRp are mainly from Glycyrrhiza uralensis (Chinese name: Gancao) and Semen armeniacae amarum (Chinese name: Kuxingren). Among them, amygdalin was selected as the optimal candidate component binds to all three key targets, and euchrenone, glycyrrhizin, and glycyrol also exhibited superior affinity interactions with ACE2, Mpro, and RdRp, respectively	Multicomponent, multitarget, and multi-approach intervention
Maiti and Banerjee [175]	2021	In silico	Bioinformatic molecular docking	Tea flavonoid catechin products	Spike glycoproteins	Epigallocatechin gallate and theaflavin gallate interact better than hydroxychloroquine	
Mahmud et al. [176]	2021	In silico	Molecular docking molecular dynamic simulation MM-GBSA scores	3063 compounds from more than 200 plants from the Asian region	Main protease	Curcumin, gartanin, robinetin	
Mahmud et al. [177]	2020	In silico	Molecular docking molecular dynamic simulation MM-GBSA scores	Plant-derived natural compounds	Main protease	Carinol, albanin, myricetin	
Mesli et al. [178]	2021	In silico	Molecular docking molecular dynamic simulations	Leaves of Corchorus olitorius Linn.	Angiotensin-converting enzyme 2	Méthyl-1,4,5-tri-O-caféoyl quinate has a stronger bond, high affinity, and gives the best docking scores compared with the co-crystallized inhibitor (PRD_002214) of the enzyme ACE2	
Mohammadi et al. [179]	2020	In silico	Molecular docking	Marine polypeptides were isolated from the hydrolysate of Pacific oyster	Main protease	The score of Leu-Leu-Glu-Tyr-Ser-Ileu ligand was higher than remdesivir	Pacific oyster compounds may have the potency to be evolved as an anti-COVID-19 main protease
Murugan et al. [180]	2020	In silico	Molecular docking molecular dynamic simulation MM-GBSA scores	Andrographis paniculata phytochemicals	3 nonstructural proteins (3 L main protease (3CLpro), papain-like proteinase (PLpro) and RNA-directed RNA polymerase (RdRp)), and a structural protein (spike protein (S))	Neoandrographolide (AGP3) has shown promising binding affinity towards all the four targets	
Naik et al. [181]	2020	In silico	Molecular docking molecular dynamic simulation ADME properties	Natural product activity and species source (NPASS) database	Endoribonuclease exoribonuclease RNA-dependent RNA polymerase (RdRp) methyltransferase and main protease	21 compounds showed maximum docking scores NPC214620, NPC52382, and NPC270578 are targeting five, four, and three-drug targets, respectively	Multitarget-based drug design
Narkhede et al. [182]	2020	In silico	Molecular docking molecular dynamic simulations	Natural products	Main protease	Glycyrrhizin, bicyclogermacrene, tryptanthrin, *β*-sitosterol, indirubin, indican, indigo, hesperetin, chrysophanic acid, rhein, berberine, and *β*-caryophyllene	Interactions with the COVID-19 main protease were highest in the case of glycyrrhizin and rhein
Nivetha et al. [183]	2020	In silico	Molecular docking molecular dynamic simulation MM-PBSA	Seselin purified from the leaf extracts of Aegle marmelos	Spike protein S2, main protease, and free enzyme of the SARS-CoV-2	Seselin had inhibitory potential over multiple SARS-CoV-2 targets	
Ogunyemi et al. [184]	2020	In silico	Molecular docking molecular dynamic simulation ADME properties	226 bioactive compounds from African herbs and medicinal plants	RNA-dependent RNA polymerase	Drugable alkaloids (10′-hydroxyusambarensine, cryptospirolepine, strychnopentamine) and flavonoids (usararotenoid A and 12*α*-epi-millettosin)	
Padhi et al. [185]	2021	In silico	Molecular docking ADME properties	415 natural metabolites isolated from several plants, fungi, and bacteria	Spike glycoprotein (S1) and the main protease	Fusaric acid, jasmonic acid, jasmonic acid methyl ester, putaminoxin, putaminoxins B and D, and stagonolide K were predicted to have considerable (ADME) and safety indices	Jasmonic acid and putaminoxins B and D bind best to main protease
Pandey et al. [186]	2020	In silico	Molecular docking molecular dynamic simulation ADME properties	10 potential naturally occurring compounds (flavonoids/non-flavonoids)	Spike glycoprotein	Fisetin, quercetin, and kaempferol consist of drug-likeness property	
Kumar et al. [187]	2020	In silico	Molecular docking	Kabasura kudineer and thonthasura kudineer are two Siddha formulations	Spike glycoprotein	Cucurbitacin B (−112.09), cardiofolioside (−111.5), apigenin (−98.84), and pyrethrin (−92.98) were observed as more effective with less bind energies	Kabasura kudineer could be a potential Siddha medicine for COVID-19
Rahman et al. [188]	2021	In silico	Molecular docking ADMET properties	Rutin	Main protease (Mpro), RNA-dependent RNA polymerase (RdRp), papain-like protease (PLpro), and spike (S) protein	Significant binding of rutin with Mpro, RdRp, PLpro, and S proteins. Main protease exhibited the strongest binding affinity	Optimal solubility, nontoxic, and noncarcinogenic properties
Rahman et al. [189]	2020	In silico	Molecular operating environment (MOE) ligand-based pharmacophore approach and a molecular docking-based screening	Natural product activity and species source (NPASS)	Type II transmembrane serine protease (TMPRSS2)	85 compounds with molecular docking comparable to or greater than that of the standard inhibitor (camostat mesylate) were identified. The top 12 compounds with the most favorable structural features were studied. The low-molecular-weight compound NPC306344 showed significant interaction with the active site residues of TMPRSS2	
Rakib et al. [190]	2020	In silico	Molecular docking	Bioactive phytocompounds isolated from Tinospora crispa	Main protease	The top nine hits might serve as potential anti-SARS-CoV-2 lead molecules, with three of them exerting biological activity	
Ramadhan et al. [191]	2020	In silico	Molecular docking	Etlingera elatior plant	Main protease	Ergosterol peroxide sitostenone	
Rangsinth et al. [192]	2021	In silico	Molecular docking ADMET properties	Natural products isolated from edible and medicinal mushrooms	Main protease	6 of 25 compounds are the best drug-like property candidates, including colossolactone VIII, colossolactone E, colossolactone G, ergosterol, heliantriol F, and velutin	
Rivero-Segura et al. [193]	2021	In silico	Molecular docking molecular dynamic simulation ADME properties	Mexican natural products	Against the SARS-CoV-2	Quercetin, riolozatrione, and cichoriin target the key proteins of SARS-CoV-2	Cichoriin reaches higher lung levels (100 mg/kg, IV); therefore, it may be considered in developing therapeutic tools
Selvaraj et al. [194]	2020	In silico	Homology modeling and molecular dynamic (MD) simulation MM/GBSA, MD simulations, and PCA calculations	Traditional Chinese medicine (TCM) database	Nsp 14 guanine-N7 methyltransferase (N7-MTase)	TCM 57025, TCM 3495, TCM 5376, TCM 20111, and TCM 31007 are the compounds from the TCM database, which can occupy and interact nicely with the substrate-binding site of N7-MTase	
Sharma and Kaur [195]	2021	In silico	Molecular docking, protein interaction calculator ADME studies	12 bioactive molecules present in essential oils of eucalyptus plant leaves	Spike (S) protein	Toruatone	
Sharma [196]	2020	In silico	Molecular docking, protein interaction calculator	Eucalyptol (1,8 cineole), an essential oil component from eucalyptus oil	Main protease	Eucalyptol may represent potential treatment potential to act as main protease inhibitor	Effective binding of eucalyptol to COVID-19 proteinase
Sharma and Kaur [197]	2020	In silico	Molecular docking, protein interaction calculator	Jensenone, an essential oil component from eucalyptus oil	Main protease	Jensenone may represent potential treatment potential to act as main protease inhibitor	
Shawan et al. [198]	2021	In silico	Pharmacophore study molecular docking molecular dynamic simulation ADME properties	43 flavonoids of 7 different classes	Against the SARS-CoV-2	Luteolin and abyssinone II were found to develop successfully docked complex within the binding sites of target proteins	
Sindhu et al. [199]	2020	In silico	Molecular docking	Clerodendrum paniculatum leaves	Main protease	Clerodol	
Singh et al. [200]	2021	In silico	Molecular docking and structural dynamic studies	Tea (Camellia sinensis) polyphenols	Nonstructural protein 16 (NSP16)	Theaflavin compound demonstrated lower binding free energy in comparison with the standard molecule sinefungin	
Singh et al. [201]	2020	In silico	Molecular docking and structural dynamic studies molecular mechanics Poisson–Boltzmann surface area (MM-PBSA) ADME properties	Polyphenols	RNA‐dependent RNA polymerase (RdRp)	EGCG, theaflavin (TF1), theaflavin-3′-O-gallate (TF2a), theaflavin-3′-gallate (TF2b), theaflavin 3,3′-digallate (TF3), hesperidin, quercetagetin, and myricetin strongly bind to the active site of RdRp	EGCG, TF2a, TF2b, and TF3 can inhibit RdRp and represent an effective therapy for COVID-19
Srimathi et al. [202]	2020	In silico	Molecular docking	Traditional herbal medicine: apo-quinine, catechin, cinchonidine, cinchonine, cupreidine, epicatechin, epiprocurcumenol, epiquinine, procurcumenol, quinidine, quinine, zedoaronediol, procurcumadiol	Against the SARS-CoV-2	Epicatechin, apo-quine	
Subbaiyan et al. [203]	2020	In silico	Molecular docking	Active constituents present in common herbs	Spike (S) protein	Epigallocatechin gallate (EGCG) was found to have the highest binding affinity with the viral S protein, followed by compounds, “F” (curcumin), “D” (apigenin), and “E” (chrysophanol)	Green tea
Surti et al. [204]	2020	In silico	Molecular docking molecular dynamic simulations	Ilimaquinone (marine sponge metabolite)	Spike receptor-binding domain, RNA-dependent RNA polymerase, Nsp10, Nsp13, Nsp14, Nsp15, Nsp16, main protease, and papain-like protease	Ilimaquinone exhibited promising inhibitory potential against all the SARS-CoV-2 target proteins, as evident from the binding energies	Most promising inhibitory candidate against the SARS-CoV-2 papain-like protease
Tao et al. [205]	2020	In silico	Network pharmacology and molecular docking.	Huashi Baidu formula (HSBDF): Chinese	Against the SARS-CoV-2	Baicalein and quercetin were the top two compounds of HSBDF, which had high affinity with ACE2	Regulate multiple signaling pathways through ACE2
Umar et al. [206]	2021	In silico	Molecular docking molecular dynamic simulation ADME properties	Azadirachta indica, Mangifera indica, and Moringa oleifera: African plants	Main protease	Most of the active phytocomponents of the study plants exhibited relative inhibitory potentials against main protease and preferred pharmacological features when compared with hydroxychloroquine	Caffeic acid, chlorogenic acid, catechin, ellagic acid, gallic acid, etc.
Umesh et al. [207]	2021	In silico	Molecular docking molecular dynamic simulation ADME properties	Chemical compounds from Indian spices	Main protease	Carnosol exhibited the highest binding affinity for arjunglucoside-I and rosmanol showed a strong and stable binding affinity with favorable ADME properties	
Yang et al. [208]	In silico	2020	High-throughput virtual screening	Natural Products Research Laboratories (NPRL)	Main protease	Curcuminoid derivatives (including NPRL334, NPRL339, NPRL342, NPRL346, NPRL407, NPRL415, NPRL420, NPRL472, and NPRL473) display strong binding affinity to COVID-19 3Lpro polyprotein	NPRL-334 revealed the strongest binding affinity
Yu et al. [209]	2020	In silico	Metascape analysis protein docking molecular docking	Mongolian medicine	SARS-CoV-2 S protein RBD domain	253 active components were predicted. Phillyrin and chlorogenic acid can effectively prevent the combination of SARS-CoV-2 S protein and ACE2 at the molecular level	
Zhang et al. [210]	2020	In silico	Molecular docking molecular dynamic simulation ADME property network pharmacology analysis	Chinese herbal medicines	Anti-2019-nCoV activity	13 compounds that exist in traditional Chinese medicines were found to have potential anti-2019-nCoV activity. 125 Chinese herbs were found to contain 2 or more of these 13 compounds. Of these 125 herbs, 26 are classically cataloged as treating viral respiratory infections	Regulating viral infection, immune/inflammation reactions, and hypoxia response

**Table 3:** Summary of other studies with in silico method.

Author	Year	Method	Detail of method	Name of compound/drug	Target	Efficacy	Comments
Abu-Melha et al. [211]	2020	In silico	Molecular docking combined with molecular dynamic simulation (MDS)	Hydrazones, pyrazoles, and pyrazines bearing thiazole moiety	Main protease	The average binding affinities of the compounds 3a, 3b, and 3c (−8.1 ± 0.33 kcal/mol, −8.0 ± 0.35 kcal/mol, and −8.2 ± 0.21 kcal/mol, respectively) are better than that of the positive control nelfinavir	
Aghaee et al. [212]	2021	In silico	Pharmacophore model molecular docking combined with molecular dynamic simulation (MDS), MM/PBSA, ADME studies	Pharmit website	Main protease	ML188, nelfinavir, lopinavir, ritonavir, and *α*-ketoamide	
Ahmad et al. [213]	2021	In silico	Structure-based virtual screening (SBVS) of ASINEX antiviral library, molecular dynamic (MD) simulations	ASINEX antiviral library	Main protease	SCHEMBL12616233, SCHEMBL18616095, and SCHEMBL20148701 compounds conformation with main protease show good stability after initial within active cavity moves, a rich intermolecular network of chemical interactions, and reliable relative and absolute binding free energies	BBB_26580140 lead and its similar analogs to be explored in vivo lead molecules
Ahmed et al. [214]	2020	In silico	Molecular docking, molecular dynamics, and structure-activity relationship	76 prescription antiviral drugs	RNA-dependent RNA polymerase (RdRp) and main protease (Mpro)	Raltegravir, simeprevir, cobicistat, and daclatasvir have higher binding energy and strong interaction with active sites of the receptor proteins (with a precision of 85%)	
Alabboud and Javadmanesh [215]	2020	In silico	Molecular docking combined with molecular dynamic simulation	88 conventional drugs, 16 vitamins, and 63 natural (plant)	Main protease	Various vitamins (B9, A, K, and E vitamins) exhibited a significantly strong interaction with the studied receptor. Pleconaril, adefovir dipivoxil, and stavudine in addition to plant-based compounds such as curcumin (Curcuma longa), anolignan A (Anogeissus acuminata), and phyllamyricin B (Phyllanthus myrtifolius) had strong ligand-protein interactions	
Alamri et al. [216]	2020	In silico	Structure-based virtual screening coupled with all-atom molecular dynamic (MD) simulations	Protease inhibitors database composed of ∼7,000 compounds	Papain-like protease	ADM_13083841, LMG_15521745, and SYN_15517940 showed stable conformation and interacted well with the active residues of papain-like protease	
Alexpandi et al. [217]	2020	In silico	Molecular docking	113 quinoline drugs	Main protease, RNA-dependent RNA polymerase (RdRp) inhibitors spike-RBD-ACE2 inhibitor	Elvitegravir and oxolinic acid are able to interact with the NTP entry channel and thus interfere with the RNA-directed 5′–3′ polymerase activity of SARS-CoV-2 RdRp. Rilapladib is the only quinoline that can interrupt the spike-RBD-ACE2 complex	Quinoline, 1,2,3,4-tetrahydro-1-[(2-phenylcyclopropyl)sulfonyl]-trans-(8CI), saquinavir, elvitegravir, oxolinic acid, and rilapladib are suggested for the treatment of COVID-19
Al‐Sehemi et al. [218]	2020	In silico	Molecular docking, MD simulation, and molecular mechanics Poisson–Boltzmann surface area (MM-PBSA) results	Phenyl furoxan, an exogenous nitric oxide donor	Main protease	Spiro-isoquinolino-piperidine-furoxan moieties can be used as an effective ligand for main protease inhibition due to the presence of key isoquinolino-piperidine skeleton with additional NO effect	
Al-Shar'i [219]	2020	In silico	Molecular docking, MD simulation, and molecular mechanics Poisson–Boltzmann surface area (MM-PBSA) results	Different databases	Main protease	9 compounds with different chemotypes	
Badavath et al. [220]	2020	In silico/in vitro	Molecular docking, molecular dynamics, and structure-activity relationship studies	Screening of 118 compounds with 16 distinct heterocyclic moieties in comparison with 5 natural products and 7 repurposed drugs	Main protease	Oxidiazoles (A2 and A4) derivatives have the best docking scores. Structure-activity relationship studies showed a good comparison with a known active main protease and repurposed drug ebselen with an IC_50_ value of −0.67 *μ*M	
Barros et al. [221]	2020	In silico	Molecular docking	24 ligands	SARS-CoV-2 receptors, Nsp9 replicase, main protease (Mpro), NSP15 endoribonuclease, and spike protein (S protein) interacting with human ACE2	Antimalarial drug metaquine and anti-HIV antiretroviral saquinavir interacted with all the studied receptors	
Basit et al. [222]	2020	In silico	Protein-protein docking and molecular dynamic simulation	Truncated version of human ACE2 (tACE2)	S Glycoprotein	tACE2 provides a high-affinity protein inhibitor for S glycoprotein	
Battisti et al. [223]	2020	In silico	Pharmacophore‐based screening, docking consensus approach (DCA), molecular dynamic simulations, common hit approach (CHA)	Aldrich Market Select (AMS) database from ChemNavigator/Sigma‐Aldrich with over 8 million unique chemical structures	Against SARS-CoV-2	10 compounds with high coronavirus inhibition potential	Lead molecules
Benítez-Cardoza and Vique-Sánchez [224]	2020	In silico	Molecular docking	500,000 compounds	Potential inhibitors of the interaction between ACE2 and SARS-CoV-2 (RBD)	20 compounds were determined by docking focused on the region of interaction between ACE2 and RBD	
Cava et al. [225]	2020	In silico	Gene Ontology and enrichment analysis protein-protein interaction (PPI) network virtual screening method	—	Against SARS-CoV-2	A protein-protein interaction network of 193 genes, 22 interactions, and 36 potential drugs for future treatment strategies including nimesulide, fluticasone propionate, thiabendazole, photofrin, and didanosine	Only didanosine is a real antiviral drug, while the others are mostly anti-inflammatory
Chen et al. [226]	2020	In silico	Crystal structure, virtual screening	7173 purchasable drugs (drugs-lib), with 4574 unique compounds and their stereoisomers	Main protease	16 candidates for consideration, ledipasvir velpatasvir	
Choudhary et al. [227]	2020	In silico	Molecular docking, MM-GBSA predictive binding energy calculations, and molecular dynamic simulation	15,754 natural and synthetic compounds	Main protease	Compound 2 (molecular bank code AAA396) and compound 3 (molecular bank code AAD146)	
Chunduru et al. [228]	2021	In silico	Molecular docking	Novel drug-like inhibitors for COVID-19	Main protease	Structure 61 was found to be more stable and can be further assessed for their antiviral activity to combat COVID-19	
Coelho et al. [229]	2020	In silico	Biochemical high-throughput screening	Compound library containing known drugs, bioactive molecules, and natural products	Main protease	Organomercuric compounds thimerosal and phenylmercuric acetate, benzophenone, Evans blue, a sulfonic acid-containing dye	
Dai et al. [230]	2020	In silico	Structure-based design	—	Main protease	Designed and synthesized two lead compounds (11a and 11b) targeting main protease. Both exhibited excellent inhibitory activity	
de lima Menezes and da Silva [231]	2020	In silico	Molecular dynamic simulations, molecular docking	DrugBank database	Nonstructural protein 1 (nsp1)	Tirilazad, phthalocyanine, and Zk-806450 showed better energy score than control molecules that have in vitro activity against nsp1 from SARS-CoV-2	Tirilazad, phthalocyanine, and Zk-806450
Debnath et al. [232]	2020	In silico	Pharmacophores studies, structure-based virtual screening, molecular dynamic (MD) simulation	Drug molecule information retrieved from DrugBank	Main protease	DB07456 and DB13592 displayed a similar type of binding interaction with co-ligands and remdesivir, and the predicted Ki values of 2 inhibitors were found to be superior to remdesivir	
Di Micco et al. [233]	2021	In silico	Molecular docking, MM-GBSA-based predictions, and molecular dynamics	Zonulin inhibitor larazotide acetate (also called AT1001)	Main protease	AT1001, besides its well-demonstrated effect in ameliorating mucosal permeability in ALI/ARDS	
El Hassab et al. [234]	2021	In silico	Structure-based virtual screening, molecular dynamic simulation, and MM-PBSA approaches	48 million drug-like compounds of the ZINC database	SARS-CoV-2 2′-O-methyltransferase (nsp16)	Compound 11 as the best potential nsp16 inhibitor herein identified, as it displayed a better stability and average binding free energy for the ligand-enzyme complex compared to sinefungin	
Elmessaoudi-Idrissi et al. [235]	2020	In silico	In silico screening, molecular docking, and dynamic approaches	5000 compounds of the ZINC database	Main protease	The prominent drug-like and potent inhibitory compounds are 2-[2-(2-aminoacetyl) aminoacetyl] amino-3-(4-hydroxyphenyl)-propanamide (ZINC000004762511), 6′-fluoroaristeromycin (ZINC000001483267), and cyclo(L-histidyl-L-histidyl) (ZINC000005116916) scaffolds	
Feitosa et al. [236]	2020	In silico	Molecular docking	—	Main protease	Melatonin can have response potential in early stages for its possible effects on ACE2 and main protease, although it is also promising in more severe stages of the disease for its action against hyper-inflammation	Do not confirm antiviral activity, but can rather be used as a basis for further preclinical and clinical trials
Gurung et al. [237]	2021	In silico	Virtual screening, molecular docking	1,36,191 molecules	Spike (S) protein receptor-binding domain (RBD) to the host cell surface receptor, angiotensin-converting enzyme 2 (ACE2)	ZINC33039472 exhibited binding free energy value lower as compared to the control (emodin) with a higher contribution by gas-phase energy and van der Waals energy to the total binding free energy	
Haider et al. [238]	2020	In silico	Computer-aided drug design (CADD) molecular docking	ZINCPharmer	Main protease	∼200 compounds were identified as having strong interaction with Mpro (ZINC20291569, ZINC90403206, ZINC95480156) that showed the highest binding energy	
Hall and Ji [239]	2020	In silico	Homology modeling molecular docking	ZINC15 database: 3447 entries	Spike glycoprotein and main protease	Zanamivir, indinavir, saquinavir, and remdesivir are among the exciting hits of main proteinase	Flavin adenine dinucleotide (FAD) adeflavin, B2 deficiency medicine, and coenzyme A, a coenzyme may also be potentially used for the treatment of SARS-CoV-2 infections
Havranek and Islam [240]	2020	In silico	Docking and molecular dynamics	2692 protease inhibitor compounds	Main protease	Phenyltriazolinones (PubChem ID: 104161460) and allosteric HCV NS5B polymerase thumb pocket 2 (PubChem ID: 163632044) have shown antiviral activity and also have high affinity towards the main protease	
Ibrahim et al. [241]	2021	In silico	Molecular dynamic simulations, molecular docking, MM-GBSA	18 anti-COVID-19 drug candidates against SARS-CoV-2 main protease	Main protease	Promising binding affinities of TMC-310911 and ritonavir	
Jaiswal and Kumar [242]	2020	In silico	Docking studies and molecular dynamic simulation	Designed a protein inhibitor	Spike (S) glycoprotein	The proposed inhibitor ΔABP-D25Y	
Jamalan et al. [243]	2021	In silico	Docking and molecular dynamic (MD) simulation	Virtual screening based on GRL-0617	Papain-like proteinase	5-(aminomethyl)-2-methyl-N-[(1R)-1-naphthalen-1-ylethyl]benzamide outperformed GRL-0617 in terms of binding affinity (−9.7 kcal/mol). 2-(4-fluorobenzyl)-5-nitro-1H-isoindole-1,3(2H)-dione, 3-nitro-N-[(1r)-1-phenylethyl]-5-(trifluoromethyl)benzamide, 5-acetamido-2-methyl-N-[(1S)-1-naphthalen-1-ylethyl]benzamide	
Jin et al. [244]	2020	In silico	Structure-assisted drug design, virtual drug screening, and high-throughput screening	10,000 compounds	Main protease	Ebselen, disulfiram, tideglusib, carmofur, shikonin, PX-12	Ebselen also exhibited promising antiviral activity in cell-based assays
Kanhed et al. [245]	2021	In silico	Systematic virtual screening approach	ASINEX BioDesign library approved drug library	Main protease	Ritonavir, nelfinavir, and saquinavir were predicted to be the most potent Mpro inhibitors. 20 molecules (pyrazoles, cyclic amides, pyrrolidine-based compounds, and miscellaneous derivatives)	
Kavitha et al. [246]	2020	In silico	Molecular docking molecular dynamic simulations	1000 protease-inhibitor-like compounds available in the ZINC database	Main protease	1,2,4 triazolo[1,5-a] pyrimidin-7-ones	
Krishnan et al. [247]	2020	In silico	Molecular docking	3978 compounds with potential antiviral activity	Endoribonuclease (NSP15)	8 compounds with good docking score and docking energy e.g., Z595015370, Z1343129850, and Z2760938911	
Kumar et al. [248]	2021	In silico	Molecular docking molecular dynamic simulation molecular mechanic Poisson–Boltzmann surface area approaches	Million molecules and natural compound databases	Main protease	Three compounds namely ZINC14732869, ZINC19774413, and ZINC19774479 displayed better binding affinities	
Kumar et al. [249]	2020	In silico	Molecular docking molecular dynamic simulations	13 approved antiviral drugs	Main protease	Indinavir was described as a lead drug. Indinavir possesses an important pharmacophore	Novel compound 16(hydroxyethylamine derivative) suitability as a strong candidate for therapeutic discovery against COVID-19
Kwarteng et al. [250]	2020	In silico	Bioinformatic approach molecular docking molecular dynamic simulations	—	Nucleocapsid (N) protein	Zidovudine triphosphate, an anti-HIV agent, as a potential inhibitor of the N-terminal domain of SARS-CoV2 N protein	
Li et al. [251]	2021	In silico	Molecular docking	21 antiviral, antifungal, and anticancer compounds	Papain-like protease	Neobavaisoflavone	
Mathpal et al. [252]	2020	In silico	Molecular docking molecular dynamic simulation MM-PBSA	3180 FDA-approved drugs from “the ZINC database”	Main protease	ZINC03831201, ZINC08101052, ZINC01482077, and ZINC03830817	
Maurya et al. [253]	2020	In silico	Molecular docking	Antiviral, anti-infectious, and anti-protease compounds	NSP10/NSP16 methyltransferase and main protease	Cyclocytidine hydrochloride, trifluridine adonitol, and meropenem penciclovir bound with a good docking score NSP10/NSP16 methyltransferase complexed with telbivudine, oxytetracycline dihydrate, methyl gallate, 2-deoxyglucose, and daphnetin	
Mohamed et al. [254]	2020	In silico	Molecular docking	12 histone deacetylases (HDACs)	Main protease	Romidepsin and its active form (RedFK)	
Monajemi and Zain [255]	2021	In silico	ONIOM (own N-layered integrated molecular orbital and molecular mechanics; QM/MM) approach	N3, ebselen, disulfiram, tideglusib, carmofur, shikonin, and PX-12	Main protease	N3, ebselen, and PX-12 inhibitors	Better inhibition from PX-12 than ebselen
Motiwale et al. [256]	2020	In silico	Molecular docking molecular dynamic simulations	Previously reported SARS-3CL protease inhibitors	Main protease	N-substituted isatin derivatives and pyrazolone	
Mutlu et al. [257]	2020	In silico	Structure-based approach molecular docking molecular dynamic simulations	FDA-approved and investigational drugs	Nsp12/Nsp8	Two drugs, RX-3117 (fluorocyclopentenyl cytosine) and nebivolol	
Naidoo et al. [258]	2020	In silico	Molecular docking molecular dynamic simulation MM-PBSA	Cyanobacterial metabolites	Main protease (Mpro) and the papain-like protease (PLpro)	Deoxycylin, drospermopsin	Cylindrospermopsin, deoxycylindrospermopsin, carrageenan, cryptophycin 52, eucapsitrione, tjipanazole, tolyporphin, and apratoxin A exhibited promising inhibitory potential against the SARS-CoV-2 Mpro. The compounds cryptophycin 1, cryptophycin 52, and deoxycylindrospermopsin were observed to display encouraging binding energy scores with the PLpro of SARS-CoV-2
Olubiy et al. [259]	2020	In silico	Molecular docking molecular dynamic simulations	Approved drugs, investigational drugs, natural products, and organic compounds	Main protease	Several tyrosine kinase inhibitors, which include a bioflavonoid and steroid hormones, bind best to main protease	Nilotinib, enasidenib, afatinib, ertapenem, phthalocyanine, hypericin, amrubicin, theacitrin A, theaflavin, amentoflavone, epigallocatechin gallate, glabrolide, cortisol, estradiol, testosterone
Özdemir et al. [260]	2020	In silico	Molecular docking density functional theory (DFT) ADME-Tox	42 coumarin derivatives	Main protease	6,7-Dihydroxy-3-phenylcoumarin derivatives gave relatively higher scores, and for all coumarins, and 4-trifluoromethylphenyl substituted coumarin had the highest score	
Ozdemir et al. [261]	2020	In silico	Molecular docking molecular mechanics Poisson–Boltzmann surface area (MM-PBSA)	Coumarin derivatives	Spike S1 subunit, NSP5, NSP12, NSP15, and NSP16	The highest score (−10.01 kcal/mol) in the coumarin group is 2-morpholinoethan-1-amine substituted coumarin	
Patel et al. [262]	2021	In silico	Pharmacophore studies	Drug library (having drugs and diagnostic agents, which are approved by FDA or other world authorities) and the ASINEX BioDesign library	Main protease	Ritonavir, nelfinavir, and saquinavir were predicted to be the most potent Mpro inhibitors 20 molecules categorized into four classes viz. disubstituted pyrazoles, cyclic amides, pyrrolidine-based compounds, and miscellaneous derivatives	
Peng et al. [263]	2020	In silico	Drug repositioning through virus-drug association prediction	—	Against SARS-CoV-2	Ribavirin was predicted to be the best small molecular drug, with a higher molecular binding energy with human angiotensin-converting enzyme 2 (ACE2), followed by remdesivir, mycophenolic acid, and chloroquine (−6.29 kcal/mol)	
Pratama et al. [264]	2020	In silico	Molecular docking	Novel 5-O-benzoylpinostrobin derivatives	Main protease	Three 5-O-benzoylpinostrobin derivatives each with fluoro, tertiary butyl, and trifluoromethyl substituents at 4-position of benzoyl group showed the lowest free energy of binding value and the highest similarity of ligand-receptor interactions with co-crystallized ligands	
Pundir et al. [265]	2020	In silico	Pharmacophore-based virtual screening molecular mechanics Poisson–Boltzmann surface area (MM-PBSA)	PubChem database	Main protease	Two compounds: PubChem3408741 and PubChem4167619 had the binding free energy of −94.02 kJ·mol^−1^ and −122.75 kJ·mol^−1^, respectively, as compared to reference X77 (−76.48 kJ·mol^−1^)	Lead molecules for targeting Mpro enzyme
Quimque et al. [266]	2020	In silico	Molecular docking molecular dynamic simulation ADME properties	97 antiviral secondary metabolites from fungi	Papain-like protease, RNA-dependent RNA polymerase, main protease, spike glycoprotein, nonstructural protein 15 (nsp15)	Two fumiquinazoline alkaloids quinadoline B (**19**), scedapin C (**15**), and polyketide isochaetochromin D1 (**8**)	Quinadoline B [19] was predicted to confer favorable ADMET values, high gastrointestinal absorptive probability, and poor blood-brain barrier crossing capacities
Rakib et al. [267]	2021	In silico	Molecular docking molecular dynamic simulation ADME properties	Selenium-containing heterocyclic compounds	Main protease	Selection of the 16 most effective selenocompounds as potential anti-COVID-19 drug candidates. Ethaselen showed potential binding affinities	
Rane et al. [268]	2020	In silico	Molecular docking molecular dynamic simulations	Diarylpyrimidine analogs	Spike glycoprotein	AP-NP (2-(2-amino-5-(naphthalen-2-yl)pyrimidin-4-yl)phenol), AP-3-OMe-Ph (2-(2-amino-5-(3-methoxyphenyl)pyrimidin-4-yl)phenol), and AP-4-Me-Ph (2-(2-amino-5-(p-tolyl) pyrimidin-4-yl)phenol) from a group of diarylpyrimidine derivatives, which appears to bind at the interface of the hACE2-S complex with low binding free energy	
Rao et al. [269]	2020	In silico	Molecular docking molecular dynamic simulations	Various small molecules	Main protease	Pyranonigrin A, a secondary fungal metabolite	
Salman et al. [270]	2020	In silico	Molecular docking molecular dynamic simulation ADME properties	Library of immunomodulatory medicinal compounds with antiviral capability	SARS proteases, spike protein, and nonstructural proteins (NSP-9, 15)	6 compounds: arzanol, ferulic acid, genistein, resveratrol, rosmanol, and thymohydroquinone	Good pharmacokinetic properties and low acute toxicity of these compounds
Sarma et al. [271]	2020	In silico	Molecular docking MM-GBSA binding free energy molecular dynamic simulations	56,079 compounds from ASINEX and Maybridge library	RNA-binding N-terminal domain (NTD) of the N protein	ZINC00003118440 is a theophylline derivative. Pyrimidone derivatives as possible inhibitors of RNA binding to the N-terminal domain of N protein of coronavirus	Lead molecules
Sepay et al. [272]	2020	In silico	Molecular docking, bioinformatics, and molecular electrostatic potential ADME studies	Benzylidenechromanones, naturally occurring oxygen heterocyclic compounds	Main protease	(Z)-3-(4 ′-chlorobenzylidene)-thiochroman-4-	Effective pharmacological properties
Shehroz et al. [273]	2020	In silico	Pharmacophore modeling molecular docking	DrugBank, ZINC, and TIMBLE databases	Spike (S) protein	Only eight molecules fit the criteria for safe oral drugs	Lead molecules
Singh and Das [274]	2021	In silico	Molecular docking	Chloroquine (CQ) hydroxychloroquine (HCQ) azithromycin	Spike (S) protein main protease host cathepsin L (CTSL) receptor-binding domain (RBD)	Azithromycin affinity scores (ΔG) with strong interactions with ACE2, CTSL, Mpro, and RBD. CQ firm bond score with Mpro HCQ and two results (ACE2 and Mpro) were firmly bound to the receptors	
Stefaniu et al. [275]	2020	In silico	Molecular docking density functional theory (DFT) computations, drug-likeness assessment	Derivatives of benzoic acid	Main protease	2,5-dihydroxybenzoic acid (gentisic acid) and octyl	A combination of the two compounds can be considered
Tachoua et al. [276]	2020	In silico	Molecular docking and structural dynamic study molecular mechanic Poisson–Boltzmann surface area (MM-PBSA) ADMET analysis	Chloroquine, quinine, nitazoxanide, doxycycline, lymecycline, cetirizine, mizolastine, indinavir	Main protease	Lymecycline mizolastine	
Uniyal et al. [277]	2020	In silico	Structure-based virtual screening molecular docking MM-GBSA binding free energy molecular dynamic simulations	Commercially available chemical libraries	Main protease	Compound AG-690/11203374_1 and AG-690/11203374_2 emerged as the best in silico hits based on the docking, MM-GBSA, dynamics, and ADMET studies	Lead molecules
Welker et al. [278]	2020	In silico/in vitro	Structure-activity relationships molecular docking fluorescence-based enzyme‐activity assay Vero E6 cells	A series of rationally designed competitive, noncovalent, nonpeptidic active site‐directed SARS‐CoV PLpro inhibitors	Papain-like cysteine proteases (PLpro)	*R*)‐5‐amino‐2‐methyl‐N‐(1‐(naphthalen‐1‐yl)ethyl) benzamide (**2b**), which is known to bind into the S3 and S4 pockets of the SARS‐CoV PLpro. Isoindoline as a new class of potent PLpro inhibitors	IC_50_ value of 2.9 ± 0.2 *μ*M
Wen et al. [279]	2021	In silico	Structure-based screening	8,820 compounds	Main protease	Trichostatin A	A histone deacetylase inhibitor and an antifungal compound
White et al. [280]	2020	In silico	Homology modeling and molecular dynamics approach molecular docking	∼970,000 chemical compounds	Helicase (Nsp13)	Nilotinib and lumacaftor have significant activity in inhibiting purified recombinant SARS-CoV-2 helicase	
Wu et al. [281]	2020	In silico	Molecular docking	11 HIV-1 protease inhibitors, 12 nucleotide-analog inhibitors, 728 approved drugs	Main protease RNA-dependent RNA polymerase	Remdesivir shows the best binding energy on RdRp and saquinavir is the best inhibitor of main protease	
Zaher et al. [282]	2020	In silico	Design, synthesis SAR study molecular docking	Newly synthesized sixteen halogenated triazole compounds	Helicase (Nsp13)	The most potent compounds were 4-(cyclopent-1-en-3-ylamino)-5-(2-(4-iodophenyl)hydrazinyl)-4H-1,2,4-triazole-3-thiol [16] and 4-(cyclopent-1-en-3-ylamino)-5-[2-(4-chlorophenyl)hydrazinyl]-4H-1,2,4-triazole-3-thiol [12]	
Zarezade et al. [283]	2021	In silico	3D-QSAR pharmacophore modeling ADMET properties, molecular docking molecular dynamic simulation MM-PBSA, hybrid QM-MM method de novo ligand design	PubChem and ZINC databases	Human angiotensin-converting enzyme 2 and main protease	ZINC12562757 and 112,260,215 were the best potential inhibitors of the ACE2 and main protease, respectively. Evo_1 compound enjoys the highest docking energy among the designed de novo ligands	Evo_1 has a stronger potential for specific inhibition of main protease, as compared to the 112, 260, 215 compound. Lead molecules

**Table 4:** Summary of studies with in vitro method.

Author	Year	Method	Detail of method	Name of compound/drug	Target	Efficacy	Comments
Agarwal et al. [284]	2020	In vitro	IL-1*β* assay using THP-1 cells, *in vivo* pharmacokinetic investigation in C57BL/6 mice	Alkenyl sulfonylurea derivatives	NLRP3 inhibition and reduction in the release of interleukin-1*β*	IL-1*β* inhibition IC_50_ of 35 nM, good oral absorption showing Cmax of 8.49 *μ*g/mL with an AUC of 48.9 *μ*g·h/mL and terminal half-life of 2.86 h, after oral route of administration at 3 mg/kg dose	Novel thiazolo-alkenyl sulfonylurea derivative **7** as potent, selective, and orally bioavailable NLRP3 inflammasome inhibitor
Akaberi et al. [285]	2020	In vitro	Vero E6 cells, RT-qPCR for the quantification of viral RNA, expression and purification of SARS-CoV-2 3CL protease in vitro enzymatic assay	NO-Donor S-nitroso-N-acetylpenicillamine (SNAP): nitric oxide	Main protease	Although the viral replication was not completely abolished (at 200 *μ*M and 400 *μ*M), SNAP delayed or completely prevented the development of viral cytopathic effect in treated cells (IC_50_ = 440.95 *μ*M ± 36.15 SE)	A dose-dependent inhibition of the 3CL protease (400 *μ*M)
Bernstein and Zhang [286]	2020	In vitro	Vero E6 cells: virus was then added at a multiplicity of infection (MOI) of 0.01, with 1 h allowed for infection. Cytotoxicity: Cell Counting Kit-8 (CCK-8) colorimetric assay	Gallium maltolate (GaM)	Inhibition of viral replication	EC_50_ (concentration producing 50% inhibition of viral replication) of about 14 *μ*M (CI 95%: 8.9–22.8 *μ*M)	No cytotoxicity was observed at concentrations up to at least 200 *μ*M
Bocci et al. [287]	2020	In vitro	Ligand-based virtual screening/Vero E6 cells. Uninfected cells and chloroquine as control inhibition of the SARS-CoV-2-mediated CPE following infection in Vero E6 cells tissue culture infectious dose (TCID_50_) assay	4000 approved drugs/glafenine, amodiaquine vorinostat, zuclopenthixol, isoxsuprine, nebivolol, ambroxol, panobinostat, and pracinostat	Inhibition of viral replication	In silico: glafenine, amodiaquine (amodiaquine), vorinostat, zuclopenthixol, isoxsuprine, nebivolol, ambroxol, panobinostat, and pracinostat. In vitro: zuclopenthixol and amodiaquine show anti-SARS-CoV-2 activity comparable with chloroquine. EC_50_ values were estimated as 0.13 *μ*M for AQ, 1.35 *μ*M for ZPX, and 2.72 *μ*M for nebivolol	Zuclopenthixol and nebivolol exhibit *in vitro* antiviral activities and potencies that are comparable or better than chloroquine and hydroxychloroquine
Cao et al. [288]	2020	In vitro	African green monkey kidney Vero E6 cells were infected with virus at an MOI of 0.01 for 1 h. Immunofluorescence assay physiologically based pharmacokinetic modeling and simulations	9 artemisinin-related compounds	Inhibition of viral replication	Arteannuin B showed the highest anti-SARS-CoV-2 potential with an EC (50) of 10.28 ± 1.12 *μ*M. Artesunate and dihydroartemisinin showed similar EC (50) values of 12.98 ± 5.30 *μ*M and 13.31 ± 1.24 *μ*M, respectively	Lumefantrine could inhibit SARS-CoV-2 *in vitro* with an EC_50_ of 23.17 ± 3.22 *μ*M. Impair viral infection by modulating host cell metabolic pathways
Chen et al. [289]	2021	In vitro	Fluorescence-based high-throughput screen, docking/pretreated with various concentrations of test compound for 1 h, followed by infection with SARS-CoV-2 (MOI of 0.0001) in the presence of test compounds, expression and purification of SARS-CoV-2 PLpro, 3CL protease from *Escherichia coli* enzymatic assay of SARS-CoV-2 PLpro cytotoxicity assay	1920 natural products	Main protease, papain-like protease	Ginkgolic acid and anacardic acid were also identified as inhibitors of 3CL^pro^, with IC_50_ values of 1.79 ± 0.58 and 2.07 ± 0.35 *μ*M, respectively, both ginkgolic acid and anacardic acid dose-dependently inhibited PLpro activity, with IC_50_ values of 16.30 ± 0.64 and 17.08 ± 1.30 *μ*M, respectively	Ginkgolic acid act as an irreversible inhibitor against both PLpro and 3CL^pro^, suggesting it is a covalent inhibitor
Gendrot et al. [290]	2020	In vitro	Vero E6 cells, replication was estimated by RT-PCR/chloroquine as control determination of the inhibition stage	Doxycycline	Inhibition of viral replication	Median effective concentration (EC_50_) and 90% effective concentration (EC_90_) for doxycycline were 4.5 ± 2.9 *μ*M and 23.5 ± 16.5 *μ*M, respectively, EC_50_ = 4.5 *μ*M	Interacted at both entry and postentry stages of the SARS-CoV-2 infection doxycycline has anti-inflammatory effects
Gupta et al. [291]	2021	In vitro	High-throughput virtual screening, molecular dynamic simulation, prime MM-GBSA/Vero E6 cells infected with the SARS-CoV-2 fluorescence-based biochemical assay for inhibitors of 3CLpro	FDA-approved drugs	Main protease, PLpro, RNA-directed RNA polymerase, helicase, NSP-15, NSP14, and 2′-O-methyltransferase	Troxerutin (main protease and PLpro) and bisindolylmaleimide derivatives (main protease and exon) had possible dual targets. Ivermectin has nonselective toxicity to the ATCC E6 Vero cells at ≤50 *μ*M and 16.67 *μ*M based on the number of nuclei counted	BIM IX specifically blocked 3CLpro *in vitro* enzymatic assay inhibition was observed with an IC_50_ value of 113.7 ± 5.2 *μ*M a known inhibitor of protein kinase C isoforms, bisindolylmaleimide IX (BIM IX), was found to be a potent inhibitor of SARS-CoV-2
Hahn et al. [292]	2020	In vitro	Vero B4 and 76 cells و Vero E6 cells/remdesivir RT-qPCR for the detection of extracellular SARS-CoV-2 in cell immunostaining for the detection of Intracellular SARS-CoV-2 Western blot analysis viral plaque and yield reduction assay neutral red assay (NRA)	(IMU-838) inhibitor of human dihydroorotate dehydrogenase (DHODH)	Inhibition of viral replication/immunomodulator	A virus-specific EC_90_ of 6.2 ± 1.9 *μ*M was measured, with no cytotoxicity observed with drug concentrations up to 100 *μ*M at 3 days p.i.	Active moiety of IMU-838, vidofludimus, possesses broad-spectrum antiviral activity. IMU-838 reduces T lymphocyte proliferation, cytokine production, and organ infiltration by leukocytes in various in vivo and in vitro models for autoimmunity
Hattori et al. [293]	2021	In vitro	VeroE6 cell-based assays with RNA-qPCR, cytopathic assays	Two small-molecule-compounds, named GRL-1720 and 5 h, containing an indoline and indole moiety	Main protease	EC(50) values of 15 ± 4 and 4.2 ± 0.7 *μ*M for GRL-1720 and 5 h	Combination of 5 h and remdesivir exhibits synergism against SARS-CoV-2.5 h might serve as a lead M (pro) inhibitor
Jang et al. [294]	2021	In vitro	Vero CCL-81 cells/Calu-3 human lung epithelial cells	Gemcitabine and its analog 2′-fluoro-2′-deoxycytidine (2FdC)	Inhibition of viral replication	50% effective concentration (EC(50)) of 1.2 *μ*M compare to remdesivir 2FdC was marginally active (EC(50) = 175.2 *μ*M)	Gemcitabine has a synergistic effect when combined with remdesivir
Jeon et al. [295]	2020	In vitro	Drug repositioning/chloroquine, lopinavir, and remdesivir were used as reference drugs/VeroE6 cell/immunofluorescence analysis	48 FDA-approved drugs	Inhibition of viral replication	Niclosamide (IC_50_, 0.28 *μ*M), ciclesonide (IC_50_, 4.33 *μ*M)	24 potential antiviral drug candidates against SARS-CoV-2 infection
Liu et al. [296]	2020	In vitro	Virtual screening/spiked pseudotyped lentivirus for imitating SARS-CoV-2 cell entry/ACE2-GFP expressing HEK293T 2 cells. HeLa Cells, 293T (human, kidney) cells, and Vero-E6 (African green monkey, kidney) cells	FDA-approved drugs	Angiotensin I-converting enzyme 2 (ACE2) and a spike protein	Nine potential candidates were selected and submitted to experimental studies. Three (romidepsin, saquinavir, and nelfinavir) of nine drugs possess the ability to suppress SARS-2-S pseudotyped particles to enter the ACE2 expressing cells in a concentration-dependent manner	Five clinical HDAC inhibitors including romidepsin, panobinostat, givinostat hydrochloride monohydrate, CAY10603, and sirtinol could inhibit noticeably the spike/ACE2-mediated cell entry of SARS-CoV-2
Ma et al. [297]	2020	In vitro	Vero E6 cells using cytopathic effect and plaque reduction assay. HCoV-229E infection in Huh-7 cells. Western blot assay	Phillyrin (KD-1) ingredient of Forsythia suspensa	Inhibition of viral replication	Inhibit SARS-CoV-2. Markedly reduce the production of pro-inflammatory cytokines (TNF-*α*, IL-6, IL-1*β*, MCP-1, and IP-10) at the mRNA levels. p-NF-*κ*B p65, NF-*κ*B p65, and p-I*κ*B*α*	KD-1 could significantly inhibit virus proliferation in vitro, the upregulated expression of pro-inflammatory cytokines induced by SARS-CoV-2
Masih et al. [298]	2020	In vitro	LPS-stimulated RAW267.4 cells by enzyme immunoassay Western blot assay	Pyrazole derivatives	Anti-inflammatory levels of interleukin-1*β*, tumor necrosis factor-*α*, and interleukin-6	Compound 6c as the most potent analog among the tested series. Inhibition of inhibitor kappa B-*α* and NF-*κ*B	
Omotuyi et al. [299]	2020	In vitro	Kinetic assay	Aframomum melegueta. 100 secondary metabolites	Main protease, 2′-O-ribose methyltransferase (NSP16), and surface glycoprotein/ACE2 receptor interface	Diarylheptanoid (letestuianin A), phenylpropanoid (4-cinnamoyl-3-hydroxy-spiro[furan-5,2′-(1′H)-indene]-1′,2,3′(2′H,5H)-trione), flavonoids (quercetin, apigenin, and tectochrysin) have been identified as high binding compounds to SARS-CoV-2 targets in a polypharmacology manner	Di-ethyl-ether (IC_50_ = 0.03 mg/L), acetone (IC_50_ = 1.564 mg/L), ethyl-acetate (IC_50_ = 0.382 mg/L), and methanol (IC_50_ = 0.438 mg/L) fractions demonstrated the best inhibition in kinetic assay
Outlaw et al. [300]	2020	In vitro	Vero E6 cell *β*-Gal complementation-based fusion assay. Viral titration and plaque reduction neutralization assay quantitative RT-PCR.	A lipopeptide that is derived from the C-terminal heptad repeat (HRC) domain of SARS-CoV-2 S	Inhibition cell-cell fusion	Inhibits cell-cell fusion mediated by SARS-CoV-2 S and blocks infection by live SARS-CoV-2 in Vero E6 cell monolayers (IC_50_) of ∼10 nM and 90% inhibitory concentration (IC_90_) of ∼100 nM	
Pitsillou et al. [301]	2021	In vitro	Molecular docking, molecular dynamic simulation/papain-like protease (SARS-CoV-2) assay kit GRL-0617 as a control enzymatic inhibition assay	300 small compounds derived predominantly from our OliveNet™ library (222 phenolics) and supplemented with synthetic and dietary compounds	Papain-like protease	Selection of 30 compounds. Hypericin possessed inhibition activity, and both rutin and cyanidin-3-O-glucoside resulted in a concentration-dependent inhibition of the PLpro	The IC_50_ values were not clarified in experiment
Pizzorno et al. [302]	2020	In vitro	Vero E6 cells/remdesivir, lopinavir, chloroquine as comparator	Favipiravir, ribavirin, umifenovir (arbidol), berberine, cyclosporine A, diltiazem	Inhibition of viral replication	Umifenovir, berberine, and cyclosporine A with estimated 50% inhibitory concentrations of 0.99, 5.2, 1.38, 3.5, 10.6, and 3 *μ*M, respectively	A strong antagonism between remdesivir and berberine, in contrast with remdesivir/diltiazem, for which we describe high levels of synergy
Plaze et al. [303]	2021	In vitro	Monkey VeroE6 cells and human alveolar basal epithelial A549-ACE2 cell RT-qPCR for the presence of SARS-CoV-2 RNA	Chlorpromazine	Inhibition of viral replication	(IC_50_) of 8.2 *μ*M in monkey VeroE6 cells (CC_50_) of 13.5 *μ*M, and selectivity index (SI) of 1.65 IC_50_ of 11.3 *μ*M, CC_50_ of 23.1 *μ*M, and SI of 2.04 in human A549-ACE2 cells	
Reznikov et al. [304]	2021	In vitro	Repurposing study molecular docking/human lung A549 cells that were transfected with hACE2 (ACE2-A549) cells, Vero E6 cells	Electronic health records of over 219,000 patients with antihistamines	Inhibition of viral replication/spike protein	IC_50_ 15.3 *μ*g/ml for hydroxyzine, 17.4 *μ*g/ml for diphenhydramine, and 2.24 *μ*g/ml for azelastine	Hydroxyzine and possibly azelastine bind angiotensin-converting enzyme-2 (ACE2) and the sigma-1 receptor as off-target disease prevention
Sakurai et al. [305]	2021	In vitro	VeroE6 cells/human colon-derived Caco-2 cells/infection assay with immunofluorescence	5-Amino levulinic acid (5-ALA)	Inhibition of viral replication	IC_50_ of 570 *μ*M/IC_50_ of 63 *μ*M in human Caco-2 cells. No significant cytotoxicity	5-ALA is synthesized in most animals and plants and we are continuously consuming it in our food. Show anti-inflammation effects in humans
Stone et al. [306]	2021	In vitro	Vero E6 monkey kidney and Calu-3 human lung adenocarcinoma cells/RT-qPCR of RNA	Stenoparib poly(ADP ribose) polymerase (PARP) inhibitor	Inhibition of viral replication	Dose-dependent activity against SARS-CoV-2 at concentrations up to 30 *μ*M with negligible cytotoxicity. Synergistic effect with remdesivir	Stenoparib impedes entry and postentry processes
Su et al. [307]	2020	In vitro	Vero E6 cells, quantitative real-time RT-PCR	Shuanghuanglian preparation, a Chinese traditional patent medicine (chlorogenic acid, phillyrin, baicalin, and baicalein)	Main protease	Dose-dependent inhibition of SARS-CoV-2 3CLpro, and the resulting IC_50_ values were 0.090, 0.064, and 0.076 *μ*L/mL for three Shuanghuanglian oral liquids produced by three different pharmaceutical companies	Baicalin and baicalein, which have good drug-like properties
Touret et al. [308]	2020	In vitro	VeroE6 cells/human colon-derived Caco-2 cell real-time RT-PCR/remdesivir as control	FDA-approved chemical library	Inhibition of viral replication	23 drugs were selected to cover the 12 different groups. 11 compounds such as macrolide antibiotics, proton pump inhibitors, antiarrhythmic agents, or CNS drugs emerged showing antiviral potency with 2 < EC50 ≤ 20 *μ*M	Two of the highest antiviral activity were obtained for azithromycin (EC50 = 2.12 *μ*M) and hydroxychloroquine (EC50 = 4.17 *μ*M). Vonoprazan and sulfadoxine show a moderate activity with EC50 above 30 *μ*M
Tree et al. [309]	2021	In vitro	Plaque inhibition assay with Vero E6 cells/differential scanning fluorimetry/ELISA assays	Unfractionated heparin (UFH), low MW heparins	Inhibition of viral replication/spike protein RBD	All the UFH preparations had potent antiviral effects, with IC_50_ values ranging between 25 and 41 *μ*g·ml^−1^, whereas LMWHs were less inhibitory by ∼150-fold (IC_50_ range 3.4–7.8 mg·ml^−1^)	Heparin directly inhibits the binding of RBD to the human ACE2 protein receptor
Vatansever et al. [310]	2021	In vitro	Docking, screening assay/Vero E6, and A549 cell culture	30 FDA/EMA-approved drugs	Main protease	Six FDA/EMA-approved drugs can potently inhibit Mpro with an IC_50_ value lower than 100 *μ*M. Bepridil exhibited strong inhibition of SARS-CoV-2 from entry and replication inside Vero E6 and A549 cells (IC_50_ = 72 *μ*M)	Bepridil indicated that it had low micromolar EC_50_ values
Wang et al. [311]	2020	In vitro	FRET-based enzyme activity assay of Mpro measurement of human TMPRSS2 activity by a FRET-based enzymatic assay surface plasmon resonance (SPR) analysis molecular docking African green monkey kidney cell Vero E6	Catechin, kaempferol, quercetin, proanthocyanidins, resveratrol, and tannic acid	TMPRSS2 (transmembrane protease serine 2) main protease	Tannic acid inhibited the activities of the two proteases with an IC_50_ of 13.4 mM for Mpro and 2.31 mM for TMPRSS2	A potent dual inhibitor
Watashi [312]	2021	In vitro	Virtual drug screening, docking/VeroE6 cells detecting the viral RNA by real-time RT-PCR or viral proteins by immunofluorescence	FDA-approved drugs	Main protease, PLpro, RdRp, helicase, spike, ACE2, and TMPRSS2	Abiraterone, amodiaquine, anidulafungin, arbidol, astemizole, atazanavir, auranofin, azithromycin bazedoxifene, bexarotene, camostat IC_50_ values <5 *μ*M	Cepharanthine, cetilistat, chloroquine, ciclesonide, cyclosporine A, digoxin, ivermectin, lopinavir, mefloquine, nelfinavir, niclosamide
Yu et al. [313]	2021	In vitro	Computer-aided drug design and biological verification surface plasmon resonance (SPR) assays and NanoBiT assays	Traditional Chinese medicine	Spike proteins	Glycyrrhizic acid, the most efficient and nontoxic broad-spectrum anticoronavirus molecule IC_50,_ was 22 *μ*M	
Zandi et al. [314]	2021	In vitro	African green monkey kidney cells (Vero CCL-81 cells) and reverse transcription-quantitative PCR (qRT-PCR). Remdesivir and *β*-D-*N*^4^-hydroxycytidine as positive drug controls	Nucleoside analogs/anti-HCV agents	Inhibition of viral replication	Sofosbuvir and favipiravir demonstrated no antiviral effect against COVID-2	
Zhang et al. [315]	2020	In vitro	Hybrid virtual screening, docking, force field-based simulation/qRT-PCR assay, indirect immunofluorescence assay (IFA) and CCK-8 assay, surface plasmon resonance (SPR) assay/Vero cells	1906 approved drugs from TargetMol-approved drug library	RNA-dependent RNA polymerase (RdRp)	Pralatrexate and azithromycin (EC_50_) values of 0.008 and 9.453 *μ*M	A new therapeutic agent pralatrexate against COVID-19 by targeting RdRp
Zhang et al. [316]	2020	In vitro	African green monkey kidney Vero E6 cell line, quantitative real-time PCR (RT-PCR) analysis, immunofluorescence microscopy, Western blot analysis	Lopinavir/ritonavir, rupintrivir, and AG7404	Inhibition of viral replication	(IC_50_) and half cytotoxic concentration (CC_50_) values of lopinavir were ∼12.01 *μ*mol/L and 80.82 *μ*mol/L; for ritonavir, the IC_50_ and CC_50_ values were 19.88 *μ*mol/L and 94.71 *μ*mol/L (SI = 4.76)	Rupintrivir inhibited SARS-CoV-2 infection only at high drug concentrations (Vero E6: IC_50_ = 34.08 *μ*mol/L, AG7404: Vero E6: IC_50_∼195.8 *μ*mol/L). Lopinavir/ritonavir should be stopped for clinical use due to the huge gap between in vitro IC_50_ and free plasma concentration
Zhu et al. [317]	2021	In vitro	Human fibroblast lung cells (MRC-5), cytopathic effect (CPE) assay, primary human airway air-liquid interface (ALI) cultures, TEER assay, CCK-8 assays	Stimulator of interferon genes (STING): dimeric amidobenzimidazole (diABZI)	Inhibition of viral replication	Potent anticoronavirus activity against both the common cold human coronavirus 229E (HCoV-229E) and SARS-CoV-2 in cell culture systems. EC_50_ of 120 *μ*M	

**Table 5:** Summary of studies with in vivo method.

Author	Year	Method	Detail of method	Name of compound/drug	Target	Efficacy	Comments
Lei et al. [318]	2020	In vivo	BALB/c mice to determine the pharmacokinetic profiles of the fusion proteins	A recombinant protein by connecting the extracellular domain of human ACE2 to the Fc region of the human immunoglobulin IgG1	Spike protein	The IC_50_ values of ACE2-Ig for SARS-CoV and SARS-CoV-2 neutralization were 0.8 and 0.1 *μ*g·ml^−1^, respectively. Desirable pharmacological properties in mice	Potential applications in the diagnosis, prophylaxis, and treatment of SARS-CoV-2
Rathnayake et al. [319]	2020	In vivo	Old male hDPP4-KI mice infected with MERS_MA_-CoV	Dipeptidyl and tripeptidyl series of compounds	Main protease	**7a**, **6c**, **7e**, **7h**, and **6j**: EC_50_ values ranging from 0.15 to 0.9 *μ*M in Vero E6 cells, IC_50_: 0.17 to 0.82 *μ*M. 40% of mice treated with compound 6h survived, and all mice treated with compound 6j were alive at the end of the study	**6j** resulted in the survival of MERS_MA_-CoV-infected hDPP4-KI mice
Schäfer et al. [320]	2020	In vivo	An immune-competent mouse model of COVID-19 by remodeling the SARS-CoV-2 S RBD at the mACE2-binding interface. Human Ace2-expressing HT1080 cells and Syrian hamster model of SARS-CoV-2	Human mAbs (hu-mAbs)	Reduction in viral load and prevent infection	Neutralizing hu-mAbs targeting SARS-CoV-2 promotes reduction in viral load and prevent infection in macaques and hamsters effective dose: 5.3 and 16 mg/kg inhibitory concentration (IC)_50_/IC90 (half-maximal/90% IC) of 4.4/18 to 26/140 ng/ml	
Schumann et al. [321]	2020	In vivo	Female C57Bl/6 mice, LPS stimulation	MP1032:5-amino-2,3-dihydro-1,4-phthalazinedione sodium salt	Immune-modulating	Direct inhibitory activity on human PARP-1 was detected in the low micromolar range (IC_50_ = 1.55 *μ*M). MP1032 works as a ROS scavenger, reduced the secretion of TNF-*α* and IL-6. MP1032 pre-treatment significantly reduced plasma cytokine levels compared with vehicle-treated mice by 50% (TNF-*α*) and 25% (IL-6). Significant reduction in SARS-CoV-2 replication	MP1032 could further attenuate prolonged virus replication by preventing oxidative stress or by limiting ADP ribosylation of the viral nucleocapsid protein via PARP-1 inhibition
Shang et al. [322]	2021	In vivo	African green female hACE2 transgenic mice, SARS-CoV-2 injection RNA extraction and qPCR, RNA in situ hybridization assay, histopathological examination	Endosomal acidification inhibitors, including chloroquine, bafilomycin A1, and NH_4_CL	Antiviral actions against SARS-CoV-2	(40 *μ*M), bafilomycin A1 (100 nM), and NH4CL (12.5 mM) suppressed the replication of SARS-CoV-2 in all cell types chloroquine (60 mg·kg^−1^) and bafilomycin A1 (0.1 mg·kg^−1^) markedly reduced virus yields in lung tissues. Alleviated viral pneumonia in hACE2 transgenic mice	Endosomal acidification inhibitors exhibited antiviral actions against SARS-CoV-2
Sheahan et al. [323]	2020	In vivo	Male and female 20- to 29-week old SPF C57BL/6J (Stock 000664 Jackson Labs) mice, whole-body plethysmography 50, 150, or 500 mg/kg EIDD-2801 2 hr prior to intranasal infection with 5*E* + 04 PFU of mouse-adapted SARS-CoV	Ribonucleoside analog *β*-d-N^4^-hydroxycytidine	Antiviral efficacy against the three most recently emerged human CoV: SARS-CoV, MERS-CoV, and SARS-CoV-2	Bioavailable NHC prodrug (*β*-d-N^4^-hydroxycytidine-5′-isopropyl ester), improved pulmonary function and reduced virus titer and body weight loss	Continued development of EIDD-2801 as a potent broad-spectrum antiviral
Tai et al. [324]	2021	In vivo	Sprague Dawley rats. (i) HCQ‐IV: 12 rats received a single dose of 0.590 mg HCQ sulfate per animal via IV injection; (ii) HCQ‐IT: 20 rats received a single dose of 0.590 mg HCQ sulfate per animal via intratracheal (IT) administration; and (iii) liposomal HCQ‐IT: 20 rats received a single dose of 0.284 mg liposomal HCQ sulfate per animal via IT administration 0.25, 1, 4, 24, and 72 hours postdose and for tissue/organ samples were 0.25, 4, 24, and 72 hours postdose	Inhalable liposomal hydroxychloroquine (HCQ)	Pharmacokinetic study	Compared with unformulated HCQ administered intravenously, liposomal HCQ showed higher (∼30-fold) lung exposure, longer (∼2.5-fold) half-life in lungs, but lower blood exposure with ∼20% of peak plasma concentration (*C*_max_) and 74% of area under the curve from 0 to 72 hours (AUC 0–72) and lower heart exposure with 23% of *C*_max_ and 58% of AUC 0–24 (normalized for dose)	Inhalable liposomal HCQ may provide clinical benefit and serve as a potential treatment of COVID-19
Tortorici et al. [325]	2020	In vivo	From two individuals recovering from severe COVID-19 disease. Surface plasmon resonance (SPR) and flow cytometry. Cell-cell fusion assay using VeroE6 cells/Syrian hamster model	Human neutralizing antibodies (S2E12 and S2M11)	Against SARS-CoV-2	IC_50_ values were 1.2 to 6.6 ng/ml to protect hamsters against SARS-CoV-2 challenge competitively block angiotensin-converting enzyme 2 (ACE2) attachment and that S2M11 also locks the spike in a closed conformation by recognition of a quaternary epitope spanning two adjacent receptor-binding domains	Antibody cocktails for prophylaxis or therapy
Wahl et al. [326]	2021	In vivo	Coronavirus replication in human lung-only mice (LoM) and histopathologic analysis LoM were administered EIDD-2801 starting 24 h or 48 h post-SARS-CoV-2 exposure and every 12 h thereafter	Ribonucleoside analog *β*-D-N^4^-hydroxycytidine (NHC): oral prodrug EIDD-2801 (also known as molnupiravir or MK-4482)	Inhibited SARS-CoV-2 replication	EIDD-2801 pre-exposure prophylaxis significantly reduced virus titers in the human lung tissues of LoM by over 100,000-fold in two independent experiments	Prophylactic administration EIDD-2801 is highly effective
Wang et al. [327]	2021	In vivo	Male hACE2 mice inoculated intranasally with SARS-CoV-2 stock virus rhesus macaque model of SARS-CoV-2 infection	Screening the FDA-approved peptide drug library with LibDock	Against SARS-CoV-2	Ten polypeptide drugs were selected. Dalbavancin showed the strongest inhibitory ability. (EC50) was ∼12 nM. Significant inhibition of SARS-CoV-2 pseudovirion entry into HEK293/hACE2 cells, with an IC_50_ of ∼53 nM in both mouse and rhesus macaque models, viral replication, and histopathological injuries caused by SARS-CoV-2 infection are significantly inhibited by dalbavancin administration	Dalbavancin directly binds to human angiotensin-converting enzyme 2 (ACE2) with high affinity, thereby blocking its interaction with the SARS-CoV-2 spike protein
Weston et al. [328]	2020	In vivo	Mice were inoculated intranasally with SARS-CoV	20 FDA-approved drugs	Against SARS-CoV-2	7 of these inhibit SARS-CoV-2 at non-cytotoxic concentrations. Many of the compounds have IC_50_ under 10 *μ*M, chloroquine and chlorpromazine did not inhibit viral replication in mouse lungs based on viral titers recovered at 4 dpi neither drug inhibited viral replication in the lungs, but both protected against clinical disease	
White et al. [329]	2021	In vivo	A human ACE2-expressing adenovirus to transduce the naturally resistant wild-type BALB/c mice and sensitize them to SARS-CoV-2 infection, K18-hACE2 mouse model	eEF1A inhibitor plitidepsin (aplidin): targeted the eukaryotic translation machinery	Inhibited SARS-CoV-2 replication	IC90 of 1.76 nM: Vero E6 IC90 of 0.88 Nm: human cell line significantly reduced genomic RNA content reduction of nearly 2 log units in SARS-CoV-2 viral titers in the lungs of the 0.3 mg/kg plitidepsin group, reduction of 2 log units in viral lung titers at day 3, similar to two daily 50 mg/kg doses of remdesivir	Plitidepsin as a host-targeted anti-SARS-CoV-2 agent with in vivo efficacy
Wu et al. [330]	2020	In vivo	10 male C57BL/6 mice related to 200 mg/kg/day GB-2 everyday by oral administration. immunohistochemistry (IHC) assessment	GB-2, the formula from Tian Shang Sheng Mu of Chiayi Puzi Peitian Temple: in Taiwan: the index compound of *Camellia sinensis* var. assamica extract	The protein and mRNA expression of ACE2 and TMPRSS2	No mouse death and no significant alteration in mouse activity or body weight (IHC) data revealed that the expression levels of both ACE2 and TMPRSS2 were markedly diminished in the GB-2 group compared with the control group 50 *μ*g/mL of theaflavin could inhibit protein expression of ACE2 and TMPRSS2	GB-2 and theaflavin could act as potential compounds for ACE2 and TMPRSS2 inhibitors
Yuen et al. [331]	2021	Ex vivo	Human lung tissues were treated with the indicated drugs overnight, followed by SARS‐CoV‐2 infection at 2 × 10^6^ PFU/well	Small molecules targeting the ULK1/Atg1 complex involved in the induction stage of autophagy (ULK1 inhibitor SBI0206965), the ATG14/Beclin1/VPS34 complex involved in the nucleation step of autophagy (class III PI3‐kinase inhibitor VPS34‐IN1), and a widely used autophagy inhibitor that persistently inhibits class I and temporary inhibits class III PI3‐kinase (3‐MA) and a clinically approved autophagy inhibitor that suppresses autophagy by inhibiting lysosomal acidification and prevents the formation of autophagolysosome (HCQ)	Target the key cellular factors involved in key steps of the autophagy pathway	Not all the tested autophagy inhibitors suppressed SARS-CoV-2 infection inhibition of class III PI3‐kinase Vps34 downstream of ULK1, in contrast to inhibition of ULK1, which caused a significant decrease in SARS‐CoV‐2 replication (EC50) of HCQ was 19 *μ*M, that of Vps34‐IN1 was 0.82 *μ*M Vps34‐IN1 potently inhibited SARS‐CoV‐2 viral replication in normal ex vivo human lung tissue culture in a dose‐dependent manner	Class III PI3-kinase may be a possible target for COVID-19 therapeutic development
Zhang et al. [332]	2021	In vitro/in vivo	Preparation of MAbs from female BALB/c mice and wild-type Balb/c mice were intranasally inoculated with hACE2-encoded adenovirus 5 (Ad5-hACE2) to allow expression of the hACE2 receptor in the lung, followed by intranasal infection with live SARS-CoV-2 3 days later	Mouse anti-SARS-CoV-2-neutralizing MAbs: 2H2 and 3C1	Spike (S) protein	(IC_50_) of 12 ng/mL effectively treat SARS-CoV-2-infected mice even when administered as late as 24 h post-infection	

## Data Availability

All data generated or analyzed during this study are included in this published article.
